# Supplementary material for: Intrinsic Curvature-Mediated Transbilayer Coupling in Asymmetric Lipid Vesicles
Source: Biophys J. 2018 Jan 9;114(1):146–57. doi: 10.1016/j.bpj.2017.11.009 (PMC5773765; doi:10.1016/j.bpj.2017.11.009)
Supplement: Document S1. Supporting Materials and Methods, Figs. S1–S10, and Tables S1 and S2 [file mmc1.pdf]

Biophysical Journal, Volume *114*

Supplemental Information

Intrinsic Curvature-Mediated Transbilayer Coupling in Asymmetric Lipid Vesicles

Barbara Eicher, Drew Marquardt, Frederick A. Heberle, Ilse Letofsky-Papst, Gerald N. Rechberger, Marie-Sousai Appavou, John Katsaras, and Georg Pabst

Supplementary Material

Instrinsic Curvature-Mediated Transbilayer Coupling in Asymmetric Lipid Vesicles

B. Eicher, D. Marquardt, F.A. Heberle, I. Letofsky-Papst, G.N. Rechberger, M.-S. Appavou, J. Katsaras, and G. Pabst

1 Sample preparation

Acceptor vesicles were prepared in 25 mM NaCl solution to a lipid concentration of 10 mg/mL. All acceptor vesicles were doped with 10 mol% POPG or POPG-d31 matching the isotopic composition of the inner leaflet hydrocarbons. Doping vesicles with PG facilitates LUV formation (see below). Throughout this work we report POPE/POPC* molar ratios, only, where the asterisk indicates the presence of POPG. During hydration samples were incubated for one hour at $\geq$ 10°C above the lipid's melting transition (T_M) with intermittent vortex mixing, followed by 5 freeze/thaw cycles of the hydrated vesicles using liquid nitrogen. Finally, LUVs were obtained by passing the lipid dispersions 31 times ($T > T_M + 10$°C) through 100 nm pore-diameter polycarbonate filters using a hand-held mini-extruder (Avanti Polar Lipids, Alabaster, AL, USA). In turn, donor vesicles (composed of outer leaflet lipids) were prepared in the form of multilamellar vesicles (MLVs) by hydrating the dry films in 20% (w/w) aqueous sucrose solution at a lipid concentration of 20 mg/mL, followed by an 1-hour incubation at $T > T_M + 10$°C, with intermittent vortex mixing, and 3 freeze/thaw cycles. Finally, donor MLVs were diluted by a factor of 20 with water and centrifuged for 30 min at 20,000 $\times g$ to remove extravesicular sucrose.

Lipid exchange was initiated by re-suspending the pellet containing donor vesicles in 35 mM mβCD (lipid:mβCD = 1:8) followed by a 2 h incubation at room temperature while being gently stirred. Next, acceptor LUVs were added to the mβCD/donor solution and stirred gently for 30 min ($T > T_M$+10°C for POPE acceptor vesicles and T = room temperature for POPC acceptor vesicles). Two different donor/acceptor molar ratios (D/A = 2 and D/A = 3) were applied yielding a different lipid exchange (see below). Dispersions containing the final aLUVs were diluted by a factor of seven with water and centrifuged at 20,000 $\times g$ for 30 min. The supernatant (containing aLUVs, residual CD and sucrose) was removed carefully and concentrated with a centrifugal ultrafiltration device (100 kDa cutoff) to <0.5 mL. Finally, sucrose and CD were removed by repeated washing in either D_2O for ^{1}H-NMR and SANS experiments, or in H_2O for all other experiments.

For control experiments, vesicles with the same but symmetric lipid distribution as aLUVs were dried down to a film under reduced atmospheric pressure using a rotary evaporator (Heidolph, Germany) with the water bath set to 40°C. The dried lipid film was redissolved in chloroform and from that point on prepared as acceptor vesicles including the extrusion step. The resulting LUVs are called 'scrambled' vesicles throughout this report.

2 Evaluation of bilayer asymmetry via ^{1}H-NMR

The paramagnetic lanthanide ion Pr^{3+} interacts with choline protons, shifting their resonance downfield as shown in (1), see Fig. S2. By adding Pr^{3+} to a vesicle suspension, the shift is selective for outer leaflet protiated choline, leading to a separate resolution of the protiated choline resonances from the inner and outer leaflet (2). The integrated area R of each resonance is proportional to the number of molecules having protiated headgroups in the corresponding leaflet (Fig. S2). The outer leaf's peak fraction is defined as:

$$f^{out} = \frac{R^{out}}{R^{in} + R^{out}}, \tag{1}$$

where the superscripts 'out' and 'in' indicate the outer and inner leaflet. When all lipids posses protiated headgroups, f^{out} directly yields the mole fraction of all bilayer lipids found in the outer leaflet:

$$X^{out} = \frac{\sum_j N_j^{out}}{\sum_j N_j} \equiv f^{out}, \tag{2}$$

where N and N^{out} denote the number of molecules in the whole bilayer and in the outer leaflet and the summation is performed over all components of the mixture. For a bilayer with an equal number of lipids in the leaflets $X^{out} = 0.5$. However, POPE and POPC have different A_L (3, 4) which means that X^{out} is defined by the area per lipid of the inner (A_L^{in}) and of the outer leaflet (A_L^{out}):

$$X^{out} = \frac{1/A_L^{out}}{1/A_L^{out} + 1/A_L^{in}} * 1.06, \tag{3}$$

where the factor 1.06 considers the slightly bigger surface of the outer to the inner leaflet (5) by assuming a vesicle size of 130 nm and a bilayer thickness of 40 Å. As only one mixture component possesses a protiated choline (POPC) we define the single-component outer leaflet peak fraction f_{PC}^{out} as:

$$f_{PC}^{out} = \frac{N_{PC}^{out}}{N_{PC}} = \frac{X^{out}\chi_{PC}^{out}}{\chi_{PC}}, \tag{4}$$

where χ_{PC}^{out} stands for the outer leaflet mole fraction of POPC. Combining the two previous equations gives the following expression for the outer leaflet mole fraction of POPC:

$$\chi_{PC}^{out} = \frac{f_{PC}^{out}\chi_{PC}}{f_{PC}^{out}}. \tag{5}$$

For a two component POPC/POPE bilayer, all compositional parameters $\chi_{PC/PE}^{out,in}$ can be expressed as:

$$\begin{array}{lcl} \chi_{PC}^{out} & = & \frac{f_{PC}^{out}\chi_{PC}}{X^{out}} \\ \chi_{PC}^{in} & = & \frac{(1-f_{PC}^{out})\chi_{PC}}{(1-X^{out})} \\ \chi_{PE}^{in(out)} & = & 1 - \chi_{PC}^{in(out)} \end{array}. \tag{6}$$

3 Supplementary Figures

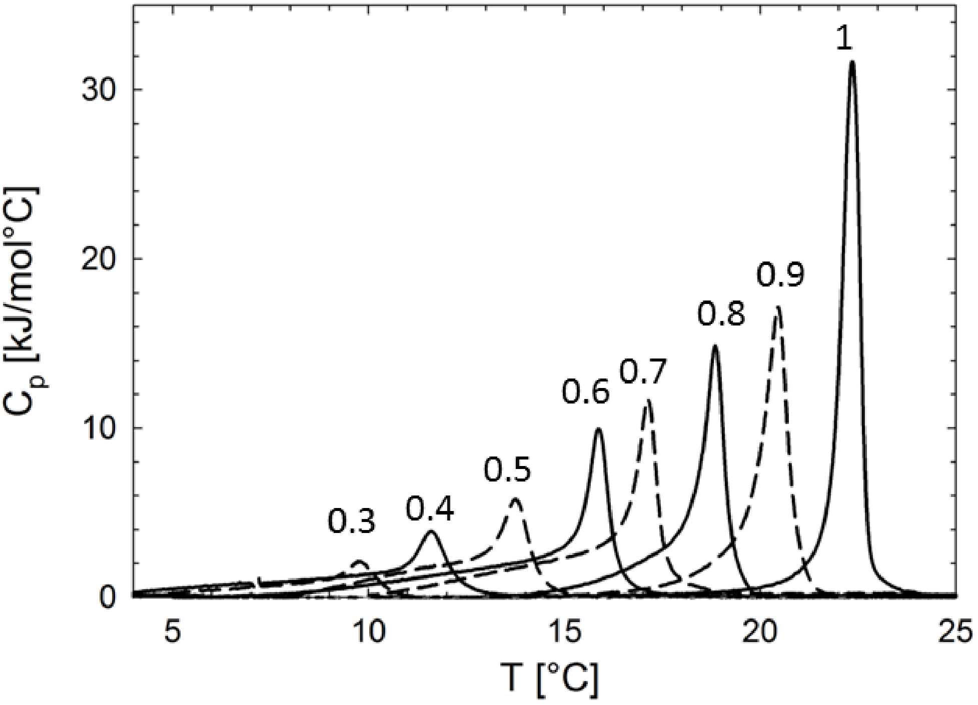

Figure S1: DSC cooling thermograms of POPE/POPC* MLV mixtures (numbers adjacent to data give the molar fractions of POPE). Note that compared to LUV data (Fig. 1) no solidus peak is visible. This relates to subtle differences in melting of LUVs and MLVs. Due to geometric constraints MLVs typically exhibit a significantly higher cooperative melting transition (see e.g. (6)). The solidus peak observed for LUVs indicates phase separation. The absence of this peak in MLV data consequently suggests that these phases are not coupled across the interstitial water layers. Note that these differences do not affect the position of the liquidus peak. DSC scans on MLVs and LUVs yield within experimental error identical T_M's (Fig. 1, insert), signifying the robustness of the applied calibration.

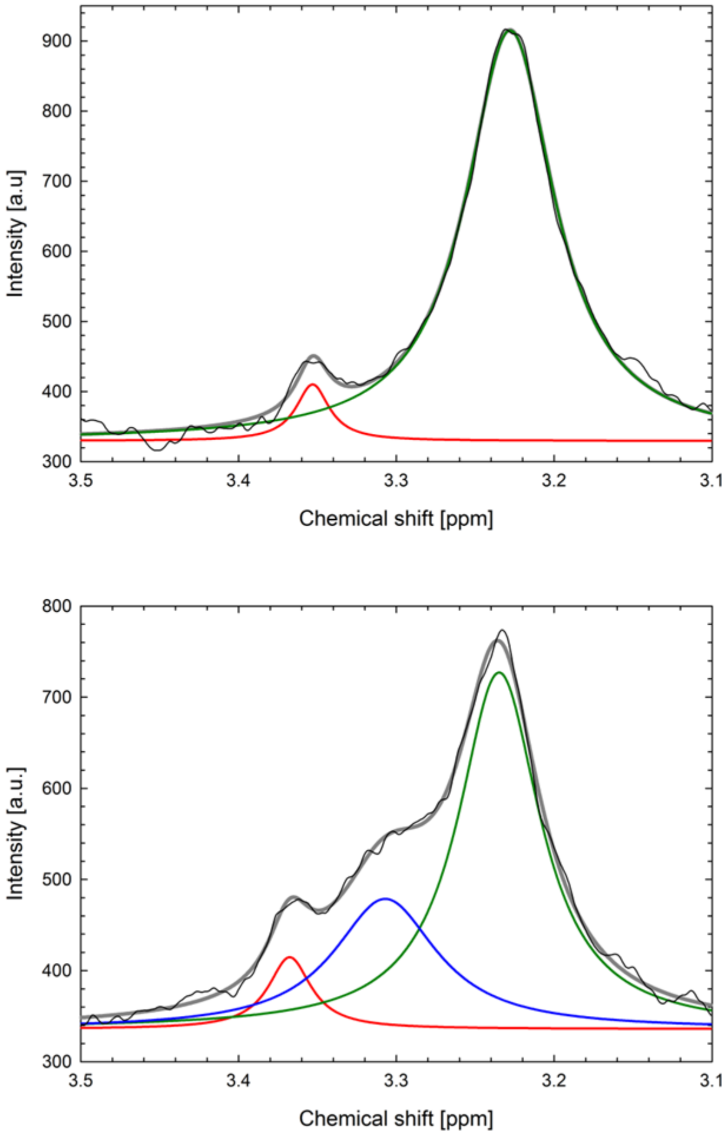

Figure S2: *Upper panel*: ^{1}H-NMR shows the choline resonance (green Lorentzian) from POPC acceptor lipids, while the red Lorentzian considers the contribution of residual mβCD. *Lower panel*: ^{1}H-NMR signal in the presence of the shift reagent Pr^{3+}. The shifted population (blue Lorentzian) relative to the unshifted population (green) reveals inner leaflet acceptor enrichment in the aLUVs.

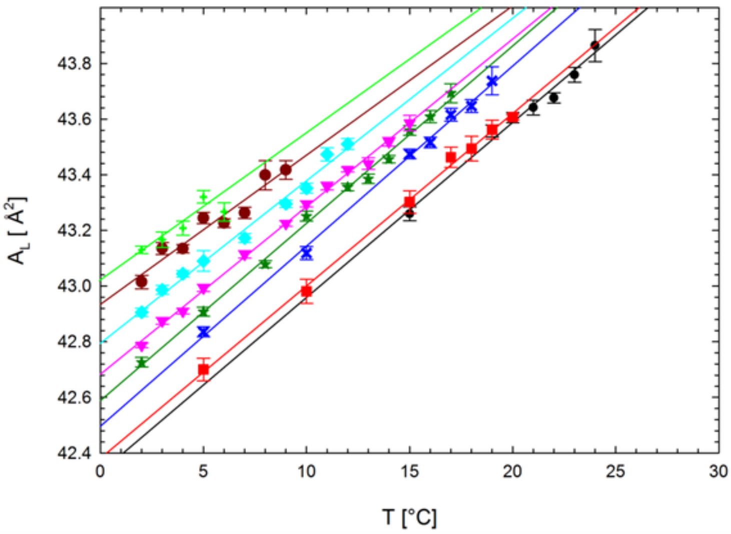

Figure S3: Temperature dependence of areas per lipid for different POPE/POPC* mixtures. The increase of POPC concentration leads to a progressive upshift of lipid areas (black line: $\chi_{POPE} = 1$, green line: $\chi_{POPE} = 0.3$)

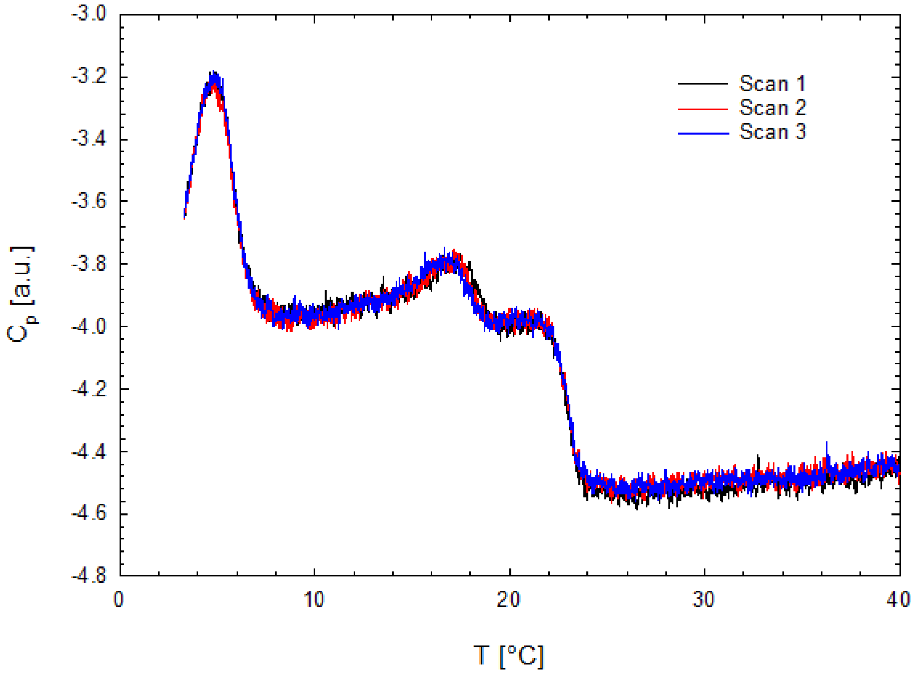

Figure S4: Three consecutive heating scans of POPE^{out}/POPC^{in} (D/A = 3). Data were background subtracted, but not normalized for sample concentration.

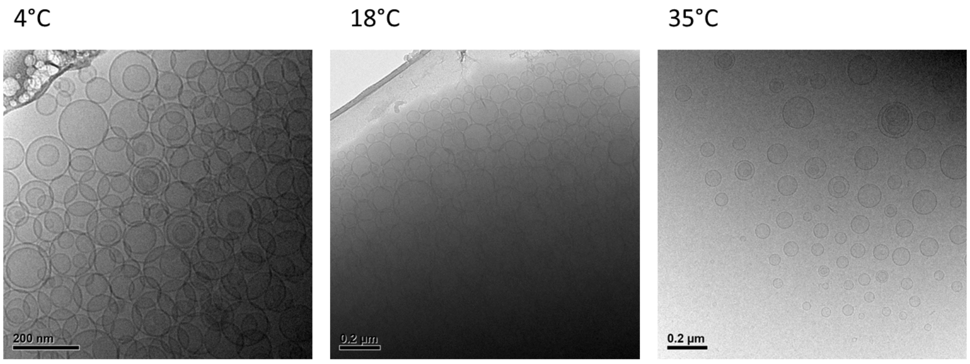

Figure S5: Cryo-TEM images of POPE^{out}/POPC^{in} aLUVs at different temperatures. The middle panel corresponds to the phase transition regime.

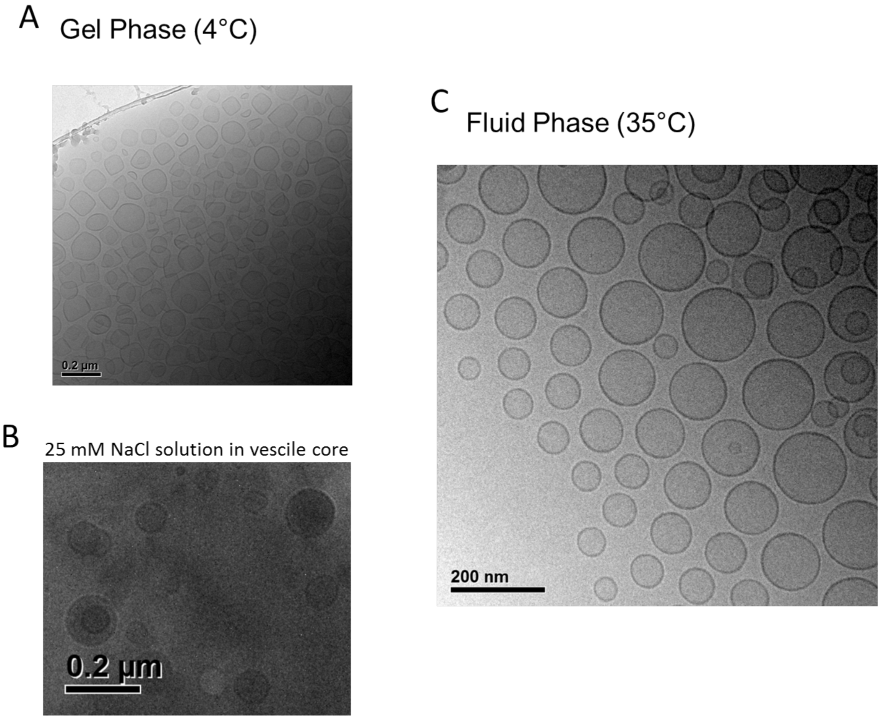

Figure S6: Cryo-TEM images of POPE* LUVs in the gel-phase without (panel A) and with a 25 mM NaCl core (panel B). Panel C shows the LUVs in fluid phase.

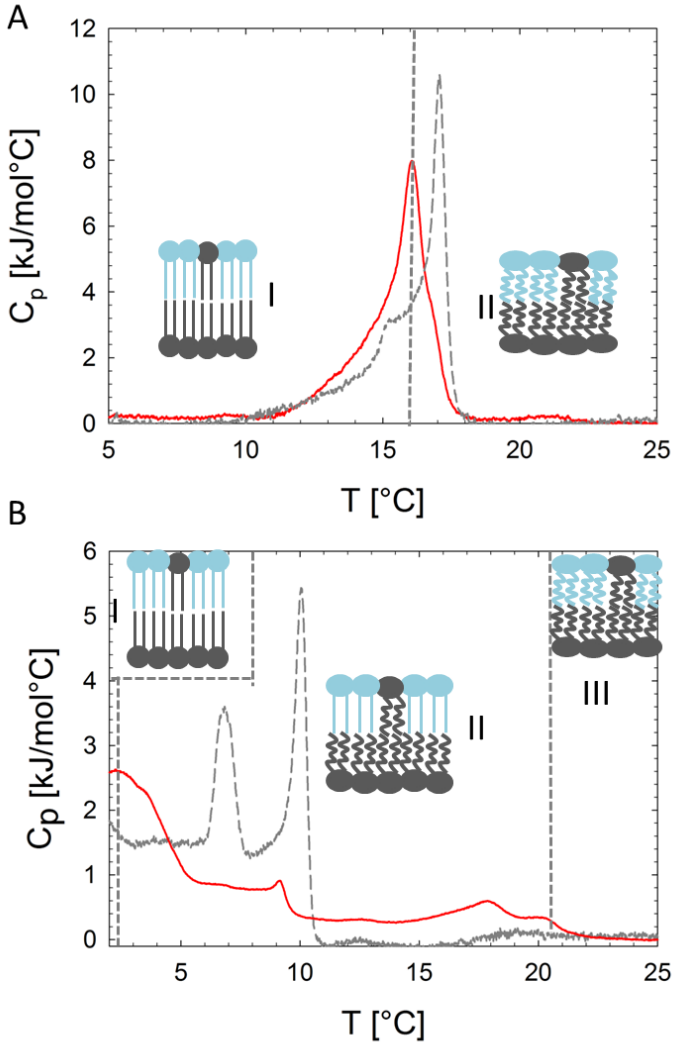

Figure S7: DSC cooling scans of POPC^{out}/POPE^{in} (panel A) and POPE^{out}/POPC^{in} (panel B) aLUVs (D/A = 2) (red lines). Transitions of corresponding scrambled LUVs are shown as gray dashed lines. Insets show schematics of leaflet structure.

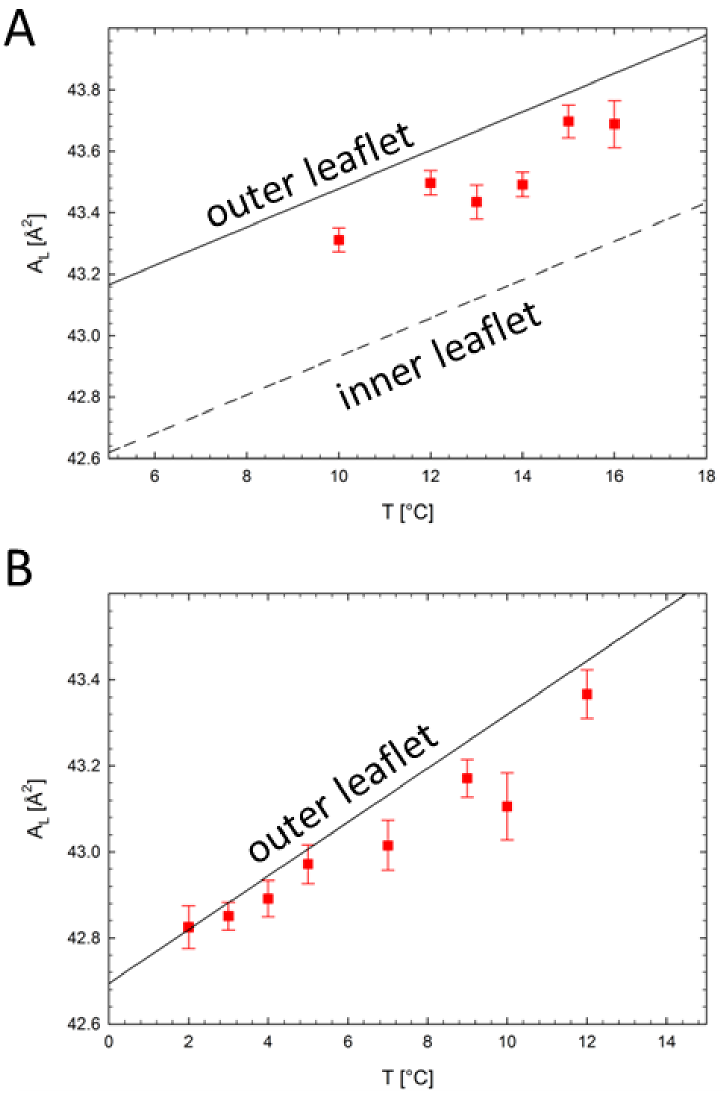

Figure S8: Area per lipid for POPC^{out}/POPE^{in} (A) and for POPE^{out}/POPC^{in} (B) as a function of temperature (symbols). Solid lines correspond to theoretical A_L's of the outer leaflet and the dashed line to theoretical A_L's of the inner leaflet calculated from the given leaflet composition.

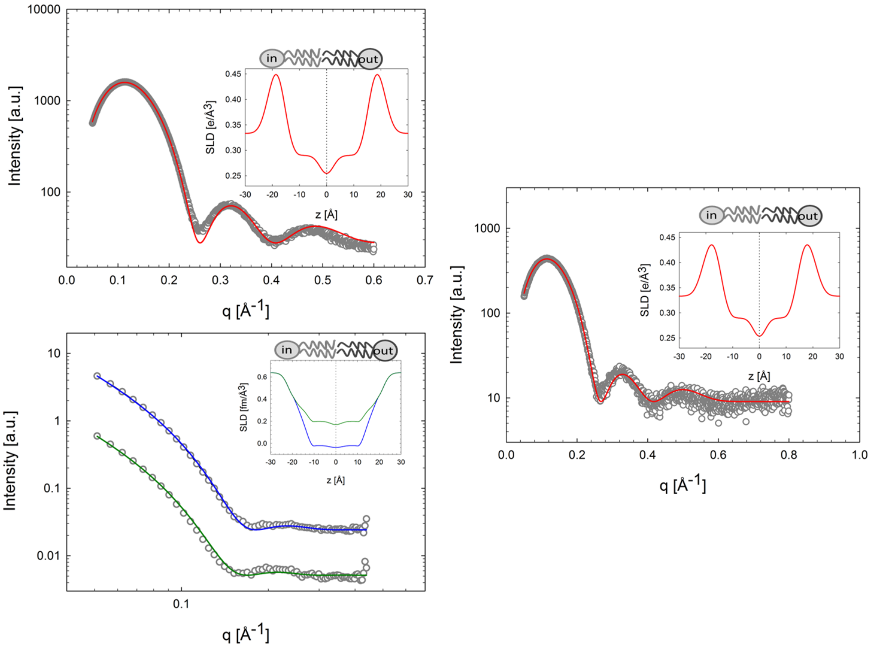

Figure S9: Analysis of scattering data of scrambled POPE/POPC LUVs (T = 35°C). *Left panel*: SAXS (*top*) and SANS (*lower*) data of POPC^{don}/POPE^{acc} LUVs. *Right panel*: SAXS data of POPE^{don}/POPC^{acc} LUVs. Solid lines correspond to best fits using the SDP model. SANS data have been obtained at two contrasts (blue line: POPC^{don}/POPE^{acc}, green line: POPC^{don}/POPE-d31^{acc}). Inserts show the corresponding scattering length density profiles.

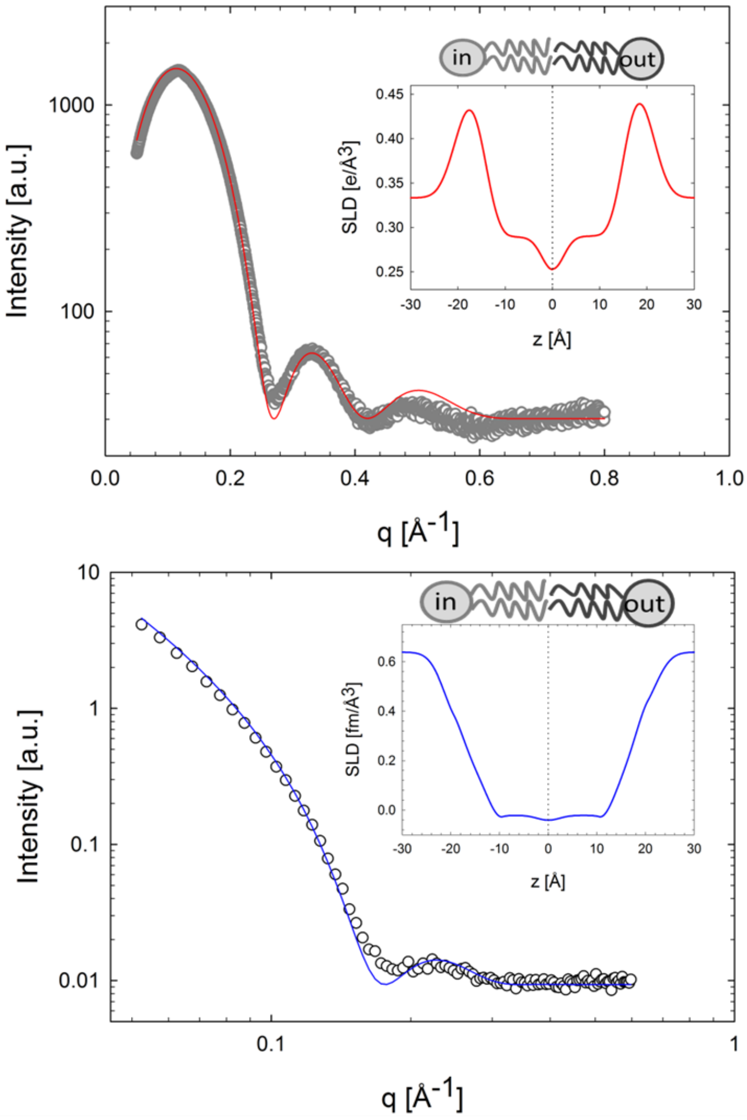

Figure S10: Joint analysis of SAXS (top panel) and SANS (lower panel) data of POPE^{out}/POPC^{in} aLUVs at 35℃. Solid lines show best fits using the aSDP model (blue line POPE^{out}/POPC^{in}). Inserts show the corresponding scattering length density profiles.

4 Supplementary Tables

Table S1: Decay of normalized bilayer asymmetry determined from ^{1}H-NMR.

	**POPE^{out}/POPC^{in}**		**POPC^{out}/POPE^{in}**	
	10°C	35°C	10°C	35°C
time (h)	ΔC	ΔC	ΔC	ΔC
0	1.00±0.04	1.00 ± 0.04	1.00 ± 0.09	1.00 ±0.08
20	1.07± 0.20	0.98 ± 0.04	1.00 ± 0.09	0.97 ± 0.07
70	1.00 ± 0.09	0.91 ± 0.05	0.94 ± 0.07	0.93 ± 0.08
118	0.96 ± 0.03	0.86 ± 0.03	0.98 ± 0.07	0.94 ± 0.05

Table S2: Structural parameters of asymmetric and scrambles POPC^{out}/POPE^{in} and POPE^{out}/POPC^{in} vesicles 35 °C determined with the aSDP-model.

	POPC^{don}/POPE^{acc}			POPE^{don}/POPC^{acc}		
	asym		scram	asym		scram
	out	in		out	in	
A_L [Å^2]	64.7	59.7	61.5	59.9	64.7	63.2
σ_{RH} [Å]	2.87	2.79	2.85	2.84	2.95	2.85
σ_{CG} [Å]	2.46	2.47	2.49	2.52	2.51	2.45
σ_M [Å]	2.00	1.99	2.04	2.03	2.01	1.97
σ_{MN} [Å]	5.01	5.01	5.01	5.01	5.01	4.95
$\|z_{RH}\|$ [Å]	19.62	20.04	19.69	20.13	18.92	19.36
$\|z_{CG}\|$ [Å]	16.35	17.56	17.12	17.02	15.88	16.21
$\|z_M\|^*$ [Å]	1.00	1.00	1.00	1.00	1.00	1.00
$\|z_{MN}\|$ [Å]	14.37	15.58	15.13	15.52	14.38	14.71

*fixed parameter

References

1. Heberle, F. A., D. Marquardt, M. Doktorova, B. Geier, R. F. Standaert, P. Heftberger, B. Kollmitzer, J. D. Nickels, R. A. Dick, G. W. Feigenson, J. Katsaras, E. London, and G. Pabst, 2016. Subnanometer Structure of an Asymmetric Model Membrane: Interleaflet Coupling Influences Domain Properties. *Langmuir* 32:5195–5200.

2. Andrews, S. B., J. W. Faller, J. M. Gilliam, and R. J. Barrnett, 1973. Lanthanide ion-induced

isotropic shifts and broadening for nuclear magnetic resonance structural analysis of model membranes. *Proc Natl Acad Sci USA* 1814–1818.

3. Kučerka, N., M. P. Nieh, and J. Katsaras, 2011. Fluid phase lipid areas and bilayer thicknesses of commonly used phosphatidylcholines as a function of temperature. *Biochim Biophys Acta* 1808:2761–2771.

4. Kučerka, N., B. van Oosten, J. Pan, F. A. Heberle, T. A. Harroun, and J. Katsaras, 2015. Molecular structures of fluid phosphatidylethanolamine bilayers obtained from simulation-to-experiment comparisons and experimental scattering density profiles. *J Phys Chem B* 119:1947–1956.

5. Marquardt, D., B. Geier, and G. Pabst, 2015. Asymmetric lipid membranes: towards more realistic model systems. *Membranes* 5:180–196.

6. Heimburg, T., 2007. Thermal Biophysics of Membranes. Wiley-VCH, Berlin.
