# Supplementary material for: Intrinsic Curvature-Mediated Transbilayer Coupling in Asymmetric Lipid Vesicles
Source: Biophys J. 2018 Jan 9;114(1):146–57. doi: 10.1016/j.bpj.2017.11.009 (PMC5773765; doi:10.1016/j.bpj.2017.11.009)
Supplement: Document S2. Article plus Supporting Material [file mmc2.pdf]

Biophysical Journal
Article

Biophysical Society

Intrinsic Curvature-Mediated Transbilayer Coupling in Asymmetric Lipid Vesicles

Barbara Eicher,[1,2] Drew Marquardt,[3] Frederick A. Heberle,[4,5,6] Ilse Letofsky-Papst,[7] Gerald N. Rechberger,[8,9] Marie-Sousai Appavou,[10,11] John Katsaras,[4,5,6] and Georg Pabst[1,2,*]

[1]University of Graz, Institute of Molecular Biosciences, Biophysics Division, NAWI Graz; [2]BioTechMed-Graz, Graz, Austria; [3]Department of Chemistry and Biochemistry, University of Windsor, Windsor, Ontario, Canada; [4]Shull Wollan Center, Oak Ridge National Laboratory, Oak Ridge, Tennessee; [5]The Bredesen Center for Interdisciplinary Research and Graduate Education, University of Tennessee, Knoxville, Tennessee; [6]Biology and Soft Matter Division, Oak Ridge National Laboratory, Oak Ridge, Tennessee; [7]Institute for Electron Microscopy and Nanoanalysis and Center for Electron Microscopy, Graz University of Technology, NAWI Graz; [8]Institute of Molecular Biosciences, University of Graz and [9]Omics Center Graz, BioTechMed-Graz, Graz, Austria; and [10]Jülich Centre for Neutron Science (JCNS) at Heinz Maier-Leibnitz Zentrum (MLZ), Forschungszentrum Jülich GmbH and [11]Forschungszentrum Jülich GmbH, Institut für Festkörperforschung, Jülich Center for Neutron Science at FRM II Outstation, Garching, Germany

ABSTRACT We measured the effect of intrinsic lipid curvature, J_0, on structural properties of asymmetric vesicles made of palmitoyl-oleoyl-phosphatidylethanolamine (POPE; $J_0 < 0$) and palmitoyl-oleoyl-phosphatidylcholine (POPC; $J_0 \sim 0$). Electron microscopy and dynamic light scattering were used to determine vesicle size and morphology, and x-ray and neutron scattering, combined with calorimetric experiments and solution NMR, yielded insights into leaflet-specific lipid packing and melting processes. Below the lipid melting temperature we observed strong interleaflet coupling in asymmetric vesicles with POPE inner bilayer leaflets and outer leaflets enriched in POPC. This lipid arrangement manifested itself by lipids melting cooperatively in both leaflets, and a rearrangement of lipid packing in both monolayers. On the other hand, no coupling was observed in vesicles with POPC inner bilayer leaflets and outer leaflets enriched in POPE. In this case, the leaflets melted independently and did not affect each other's acyl chain packing. Furthermore, we found no evidence for transbilayer structural coupling above the melting temperature of either sample preparation. Our results are consistent with the energetically preferred location of POPE residing in the inner leaflet, where it also resides in natural membranes, most likely causing the coupling of both leaflets. The loss of this coupling in the fluid bilayers is most likely the result of entropic contributions.

INTRODUCTION

Lipid asymmetry is a hallmark of biological membranes (1,2). In particular, prototypical mammalian plasma membranes are known to be composed of an outer leaflet enriched in high-melting lipids, such as sphingomyelin (SM) and phosphatidylcholine (PC), whereas phosphatidylserine (PS) and phosphatidylethanolamine (PE) lipids are predominantly located in the inner leaflet (3,4). However, the preferred location of cholesterol—the most abundant lipid of mammalian plasma membranes—is still a matter of dispute (5–7).

One of the enduring questions concerning plasma membrane architecture and lipid asymmetry is the possibility of bilayer leaflets coupled to each other. This coupling may influence a number of physiological processes that require communication between, for example, receptors secreted to the exoplasm and components of signal transduction pathways in the cytoplasm (8). It is particularly intriguing that the lipid composition of the outer leaflet favors the formation of raft-like domains (9,10), whereas that of the inner leaflet does not (11). Theoretical treatments have considered a coupling related to intrinsic lipid curvature (12,13), headgroup electrostatics, cholesterol flip-flop, and dynamic chain interdigitation (14,15), or thermal membrane fluctuations (16), implying that interleaflet coupling does not require (nor does it exclude) contributions from proteins.

About a decade ago, experimental evidence of transbilayer domain coupling was obtained using planar bilayers, where domains in one leaflet induced lipid ordering and the formation of domains in the apposing leaflet (17–19). Furthermore, the coupling strength increased with the chain melting temperature of the lipids in the distal leaflet (18), a

Submitted September 13, 2017, and accepted for publication November 9, 2017.

*Correspondence: georg.pabst@uni-graz.at

Editor: Tommy Nylander.

https://doi.org/10.1016/j.bpj.2017.11.009

© 2017 Biophysical Society.

This is an open access article under the CC BY license (http://creativecommons.org/licenses/by/4.0/).

finding that was reproduced by MD simulations (20). A coarse-grained lipid simulation also found that transbilayer coupling affects rotational and lateral lipid diffusion dynamics (21).

In symmetric, solid supported membranes with coexisting fluid lipid domains, a significant threshold for shear stress was reported for moving like domains out of register. This finding is an indication of strong transbilayer coupling of the domains (22). However, no dependence on the hydrocarbon length was observed in similar experiments (23), suggesting that dynamic (partial) chain interdigidation does not provide a significant contribution to interleaflet coupling. Other reports on solid supported asymmetric bilayers did not find domain registration (24,25). However, this may be partially related to subtleties in the preparation of planar membranes that can lead to a rapid loss of asymmetry (26,27). Hence, free-floating asymmetric lipid vesicles exhibiting slow lipid flip-flop (28), which can reliably be fabricated (29,30), appear to be excellent systems for the study of transbilayer coupling mechanisms.

Asymmetric lipid vesicles with their outer leaflets enriched in SM and with inner leaflets composed of monounsaturated PC, PS, disaturated PC, and PS/PE mixtures melted independently of one another (29,31). However, increased order of the inner fluid monolayer in the presence of a gel outer leaflet showed a weak coupling (31). In the case of asymmetric vesicles with mixed-chain lipids in one leaflet, it was noted that there was a slowing down of lateral diffusion in the apposing leaflet due to partial chain interdigitation (32). Interestingly, this did not affect the overall lipid chain order.

Recently, we performed small-angle neutron and x-ray scattering (SANS and SAXS, respectively) experiments on asymmetric vesicles made of PCs, and observed significant changes in the packing of outer-leaflet gel domains enriched in dipalmitoyl-phosphatidylcholine (DPPC), as a result of the fluid inner leaflet composed of palmitoyl-oleoyl-phosphatidylcholine (POPC) (30). This effect disappeared when both leaflets were in the fluid phase (33).

This work focuses on the "sidedness" of transmembrane coupling in asymmetric large unilamellar vesicles (aLUVs). In particular, we fabricated aLUVs composed of POPC and palmitoyl-oleoyl-phosphatidylethanolamine (POPE) with either $POPC^{out}/POPE^{in}$, or $POPE^{out}/POPC^{in}$ asymmetry, where the superscripts "in" and "out" refer to the inner and outer bilayer leaflet, respectively. Combining the data from different techniques, i.e., SAXS, SANS, wide-angle x-ray scattering (WAXS), differential scanning calorimetry (DSC), dynamic light scattering (DLS), and cryo-transmission electron microscopy (TEM), we observed strongly coupled leaflets when the inner leaflet was made up of only POPE. This coupling was manifested by a single melting transition and a similar acyl chain packing in both leaflets. In turn, aLUVs with reversed asymmetry (i.e., POPC in the inner leaflet) exhibited a broad melting transition, indicative of largely decoupled monolayers. These data provide evidence for an intrinsic curvature-mediated mechanism that energetically favors POPE—a lipid which has a significant negative intrinsic curvature—to be located in the inner leaflet. Further, we found no evidence for transbilayer coupling in fluid-phase bilayers regardless of POPE's sidedness, indicating that the loss of coupling is most likely due to entropic contributions, and that neither intrinsic curvature nor partial chain interdigitation play a significant role.

MATERIALS AND METHODS

Sample preparation

POPC, POPE, and palmitoyl-phosphatidylglycerol (POPG), including chain perdeuterated POPE-d31 and POPG-d31, were obtained from Avanti Polar Lipids (Alabaster, AL) and used without further purification. D_2O was purchased from Euroiso-top (Saarbrücken, Germany) and methyl-β-cyclodextrin (mβCD) from Sigma-Aldrich (Vienna, Austria). Purified water (18 MΩ/cm) was obtained using Purelab UHQ (Elga Labwater, Woodridge, IL). Lipid stock solutions were prepared by dissolving weighed amounts of dry lipid powder in chloroform and assayed for lipid concentration to within 1% uncertainty using standard procedures (34). Appropriate volumes were taken from stock solutions, dried under a stream of nitrogen, and then placed under vacuum for at least 12 h to remove residual organic solvent.

Fabrication of aLUVs followed a previously established protocol involving CD-mediated lipid exchange between acceptor and donor vesicles (30) (for details, see the Supporting Material). For control experiments, we prepared vesicles with the same, but symmetric lipid distribution (see the Supporting Material), which we denote as "scrambled" vesicles throughout this report. Further, we prepared symmetric LUVs with known POPE/POPC composition for DSC and WAXS calibration experiments (see below) by mixing appropriate amounts of organic lipid stock solution. These samples also contained 10 mol % POPG, which is indicated by the asterisk in the reported POPE/POPC* molar ratios. The protocol for obtaining LUVs from these samples was identical to that applied for acceptor vesicles.

Exchange efficiency and lipid distribution: DSC

DSC experiments were performed on a MicroCal VP-DSC high-sensitivity DSC (MicroCal, Northhampton, MA) at a scan rate of 30°C/h. Data were used: 1) to determine the total lipid exchange achieved; and 2) to measure the thermotropic behavior of the aLUVs. Data were corrected for sample concentration, and background was subtracted using a linear baseline (MicroCal Origin).

Symmetric LUVs prepared at various POPE/POPC* molar ratios showed thermograms typical for binary lipid mixtures with a liquidus peak at T_M and a solidus peak that became more prominent with increasing POPC concentration (Fig. 1). Note that doping POPE with 10 mol % POPG lowers the T_M by ~1.0°C (35). Throughout this work, only cooling scans were considered. Furthermore, the lowest POPE fraction, χ_{POPE}, measured was 0.3. These concentrations were arrived at by considering the low melting transition of POPC (~ −3.5°C (36)) and instrumental capability, which did not allow us to measure below 2°C. Due to hysteresis effects, cooling scans report a T_M that is ~1.2–2.0°C lower than for heating scans. The presented analysis was performed on the second cooling scan for each sample.

The obtained T_M values were found to increase linearly with POPE concentration in the studied compositional range ($T_M = a_0 + a_1\chi_{POPE}$), with $a_0 = 4.2 \pm 0.5$°C and $a_1 = 18.2 \pm 0.2$°C (Fig. 1, *inset*). This allowed us to determine lipid exchange from the DSC data of scrambled LUVs.

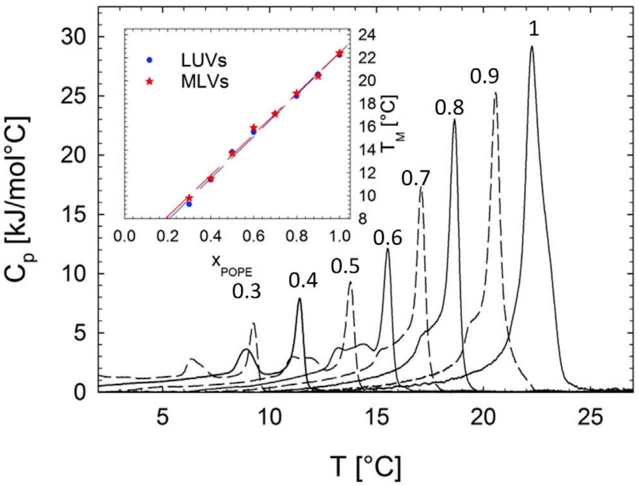

FIGURE 1 DSC cooling thermograms of POPE/POPC* mixtures. Numbers adjacent to the heat-capacity (c_P) maxima indicate the given χ_{POPE}. The inset shows the concentration dependence of the T_M values of LUVs and MLVs, which served as controls (Fig. S2). To see this figure in color, go online.

Exchange efficiency and lipid distribution: ultra-performance liquid chromatography-mass spectrometry

Alternatively, lipid exchange was determined by ultra-performance liquid chromatography-tandem mass spectrometry (UPLC-MS) as described previously (33). UPLC-MS measurements were conducted with an AQUITY-UPLC system (Waters, Manchester, UK) equipped with a BEH-C18-column (2.1 × 150 mm, 1.7 μm) (Waters) used for sample separation (37). A SYNAPTG1 qTOF HD mass spectrometer (Waters) equipped with an electrospray ionization source was used for analysis. Data acquisition was done by MassLynx 4.1 software (Waters), and for lipid analysis, the "Lipid Data Analyzer" software (38) was used. For UPLC-MS, the mole fraction, χ_i, of a single component can be determined directly using

$$\chi_i = \frac{P_i}{\sum_i P_i}, \tag{1}$$

where P_i is the area of the ith lipid peak. This relationship is strictly valid when the lipid peak area fractions vary linearly with mixture composition. UPLC-MS data was corrected using a calibration curve of 1:1:1 molar ratio of POPE/POPC/POPG measured at concentrations between 0.1 and 100 μg. Parameter uncertainties were estimated to be <5%.

Exchange efficiency and lipid distribution: solution NMR

^{1}H-NMR was used to determine the degree of asymmetry following previously published protocols (30). Briefly, ^{1}H-NMR spectra were collected on the Avance III 300 or 400 MHz spectrometers (Bruker, Billerica, MA) using Bruker TopSpin acquisition software, and were processed with TopSpin 3.2. A standard ^{1}H pulse sequence with a 30° flip angle and a 2 s delay time were used to collect 32 transients at 35 and 50°C. Data were processed with a line-broadening parameter of 2 Hz.

The outer-leaflet fraction of POPC f_{PC}^{out} relative to the inner leaflet was determined by quantifying the shifted versus the non-shifted choline resonance intensities after addition of 1 μL of a 1 mM $Pr(NO_3)_3$ $6H_2O$ (Pr^{3+}) solution (see Fig. S2 for further details). Data were averaged over three consecutive measurements. Combined with the total fraction of POPC (χ_{POPC}), which was determined by the above detailed exchange efficiency assay, the mole fraction of POPC in each leaflet is determined from

$$\chi_{PC}^{out} = \frac{f_{PC}^{out}\,\chi_{POPC}}{X^{out}}, \tag{2}$$

where X^{out} corresponds to the fractional number difference of outer- and inner-leaflet lipids due to vesicle geometry (see the Supporting Material for further details). Complementing POPE and POPC leaflet compositions were derived from $\chi_{PE}^{out/in} = 1 - \chi_{PC}^{out/in}$ and $f_{PC}^{in} = 1 - f_{PC}^{out}$.

Similar experiments allowed assessment of aLUV stability by following the decay of shifted choline resonance intensity (28). Specifically, passive lipid transbilayer diffusion rates were derived using

$$\Delta C = \frac{2f_{PC}^{out} - 1}{2f_{PC,0}^{out} - 1}, \tag{3}$$

where $f_{PC,0}^{out}$ is the fraction of POPC in the outer leaflet at time zero, i.e., immediately after aLUV preparation. Note that these measurements were taken on aliquots of aLUVs incubated at a given temperature, where Pr^{3+} was added immediately before each NMR scan.

Vesicle size and morphology: DLS

Vesicle size was measured by DLS using a ZetasizeNANO ZSP (Malvern Instruments, Malvern, United Kingdom) equipped with a 10 mW laser with $\lambda = 632.8$ nm. Measurements were conducted in glass cuvettes at a fixed measurement angle of 173°. At each temperature, samples were equilibrated for 5 min before the start of an experiment. We report averaged values from three consecutive measurements, each consisting of 15 frames (exposure time, 10 s) as well as the polydispersity index (PDI = $(\text{width}/\text{size})^2$).

Vesicle size and morphology: TEM

All TEM images were recorded with a Gatan system mounted on a Tecnai12 electron microscope (FEI Company, Hillsboro, OR), equipped with a LaB_6 filament operating at 120 kV. Electron micrographs were recorded on a Gatan Bioscan CCD 1 × 1 k camera. A Leica EM GP grid plunger, which allowed temperature control between 4 and 60°C and a relative humidity of 99% was used to spot samples on EM support grids (holey carbon film on copper grid). After carefully blotting the excess sample with filter paper, TEM grids were plunged rapidly into liquid ethane to prevent the formation of ice crystals. Samples were subsequently stored in liquid nitrogen until needed.

Membrane structural parameters: gel domains/leaflets—WAXS

WAXS experiments were performed using SAXSpace (Anton Paar, Graz, Austria) equipped with an Eiger R 1 M detector system (Dectris, Baden-Daettwil, Switzerland) and a 30 W-Genix 3D microfocus x-ray generator (Xenocs, Sassenage, France) supplying Cu-Kα ($\lambda = 1.54$ Å) radiation. WAXS was recorded by setting the sample-to-detector distance (SDD) to 180 mm.

All samples were taken up in μ-cell glass capillaries (diameter, 1 μm; Anton Paar) and equilibrated for 10 min at each temperature and to within ±0.1°C using a Peltier stage (TC Stage 150, Anton Paar). The exposure time was set to 1 h (six frames, each 10 min long). Data integration was performed using SAXStreat (Anton Paar). Background scattering originating from water and the glass capillary was subtracted after smoothing using the ATSAS suite (39).

WAXS data analysis was performed in the range $q = 1.3 - 1.6$ Å^{-1}. In the gel phase, the acyl chains of the studied lipid mixtures pack in a two-dimensional hexagonal lattice, allowing us to calculate the area per lipid directly from the position (q_{11}) of the chain-chain correlation peak (40)

$$A_L = \frac{16\pi^2}{\sqrt{3}q_{11}^2}. \quad (4)$$

To disentangle the POPE* and POPC* A_L's, a series of WAXS experiments were performed on the same symmetric lipid mixtures studied by DSC (see above). The resulting data (Fig. S3) can be collapsed on a single curve using reduced temperatures ($T - T_M$), where T_M was determined from DSC data (Fig. 2).

A linear regression ($A_L = k \times (T - T_M) + A_{POPE}^{T_M}$) yielded $k = 0.0625 \pm 0.0002$ Å^2/°C and $A_{POPE}^{T_M} = 43.6 \pm 0.002$ Å^2. The latter value corresponds to the area per lipid of POPE* at T_M. Note that due to the presence of POPG, this value is ~0.8 Å^2 higher than that of pure POPE (35,41). Assuming linear additivity, the apparent area per POPC* molecule at a given temperature in the gel phase is then derived from

$$A_{POPC} = \frac{A_L - \chi_{POPE}A_{POPE}}{1 - \chi_{POPE}}, \quad (5)$$

where A_{POPE} is the measured area per lipid of POPE* taken from the linear regression (Fig. 2). This allowed us to calculate the average A_L of any (symmetric) POPE/POPC* mixture below its T_M.

Furthermore, the lateral correlation parameter of scattering domains, ξ_D, which is a measure for the size of gel domains, was estimated using the Scherrer equation

$$\xi_D \simeq \frac{\lambda}{\beta \cos(\theta)}, \quad (6)$$

where β is the full width at half maximum of the chain correlation peak corrected for instrumental broadening, and θ is the Bragg angle.

Membrane structural parameters: fluid leaflets—SAXS/SANS

SAXS experiments were performed at the P12 BioSAXS beamline located at PETRA III (European Molecular Biology Lab/Deutsches Elektronen Synchrotron) storage ring in Hamburg (42). Samples were exposed to a total photon flux of 5×10^{12} s^{-1} at 20 keV with an x-ray beam focused to 120 × 120 μm. Data were collected by a Pilatus 2 M detector (Dectris) at SDD = 3.1 m. For measurements, samples were transferred into temperature-controlled multi-well plates and equilibrated for 10 min at a specified temperature. A robot delivered 20–35 μL of the sample into a pre-heated glass capillary. For each sample, 20 frames were recorded with an exposure time of 0.045 s. Background was measured before and after each sample exposure. To detect possible radiation damage, data collected in subsequent frames were compared using a standard F-test (43). Primary data treatment was performed using ATSAS (39).

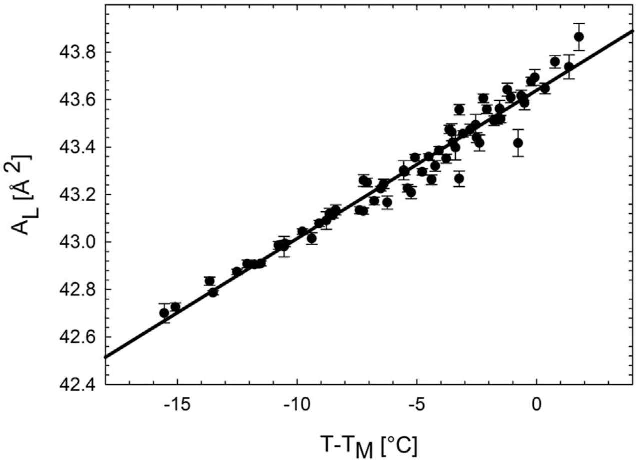

FIGURE 2 Gel area-per-lipid (A_L) calibration curve determined from WAXS. Data on an absolute temperature scale are shown in Fig. S8.

Neutron-scattering experiments were performed at KWS-1 (FRM II, Munich-Garching, Germany) (44,45) and at the BL-6 extended Q-range SANS (EQ-SANS) instrument of the Spallation Neutron Source at Oak Ridge National Laboratory. Samples were loaded into 1-mm path length 404,000-QX quartz cuvettes (Hellma, Jena, Germany) or 1 mm banjo cells, and mounted in a temperature-controlled holder ($\Delta T \sim \pm 1$°C). Typical measurement times were 30 min. At KWS-1, data were obtained with a two-dimensional scintillation detector using neutrons of $\lambda = 5$ Å (wavelength spread, $\Delta\lambda/\lambda = 0.1$) at SDDs of 1.21 m and 7.71 m, yielding a total q-range of 0.005–0.42 Å^{-1}. Data were corrected for detector pixel sensitivity, dark current, sample transmission, and background scattering from D_2O using the QTIKWS software from JCNS (Garching, Germany). EQ-SANS data were measured at two SDDs, 1.3 and 4.0 m, using wavelength bands of $\lambda = 4.0$–7.5 Å and $\lambda = 10.0$–13.5 Å, corresponding to a q-range of 0.005–0.5 Å^{-1} Data were collected with a two-dimensional ^{3}He position-sensitive detector and reduced to one-dimensional $I(q)$ scattering curves using the Mantid software (46).

Structural parameters of each leaflet were determined by a joint analysis of SANS and SAXS data using the asymmetric scattering density (aSDP) model (33). Briefly, the scattered intensity (SAXS or SANS) of aLUVs $I(q)$ can be well approximated for sufficiently dilute systems and for $q > 0.03$ by

$$I(q) \approx |F_{FB}(q)|^2, \quad (7)$$

where $|F_{FB}|^2$ is the flat-bilayer form factor, which contains information about the distribution of matter across the bilayer (47,48). The aSDP model describes bilayer structure in terms of one-dimensional volume probability profiles (VPPs) of quasi molecular lipid fragments. Specifically, each leaflet was parsed into methyl (M), hydrocarbon (HC), carbonyl + glycerol (CG), and residual headgroups (RHs). The latter group contains the choline methyl + phosphate groups in the case of PC, and $CH_2CH_2NH_3$ + phosphate groups in the case of PE. To reduce the number of adjustable parameters, a single Gaussian was used to describe the RH group in each leaflet. The corresponding scattering-length densities and volumes were derived from molecular averages according to the leaflet composition using previously reported data (49,50). Further, the effect of rapid hydrogen/deuterium exchange occurring in the primary amines of PE headgroups was taken into account for SANS data analysis (50,51). Similar to the procedure for RH, the VPPs of the M and CG groups were also modeled by Gaussians, whereas smooth plateau-like functions were used to describe the HC groups (33).

The lateral area per lipid in each leaflet was calculated using

$$A_L^{out,\,in} = \frac{V_C^{out,\,in}}{D_C^{out,\,in}}, \quad (8)$$

where V_C is the acyl chain volume including M and HC groups, and D_C is the hydrocarbon chain length given by the distance between the bilayer center and the 50%-probability value of the HC group. All SAXS/SANS data were analyzed jointly, i.e., using a single optimization procedure. For further details of the aSDP model and data analysis procedures, see (33).

RESULTS

Characterization of aLUV composition

To address the "sidedness" question, we fabricated aLUVs with POPE acceptor and POPC donor vesicles, denoted as $POPC^{out}/POPE^{in}$, as well as POPC acceptor and POPE donor vesicles, denoted as $POPE^{out}/POPC^{in}$. For each system, two batches with different donor/acceptor (D/A) ratios were prepared and assessed for their composition as detailed in a previous section.

The resulting leaflet compositions are presented in Table 1. Interestingly, we also found donor lipid in the inner leaflet of aLUVs. This may be partially due to the presence of residual small unilamellar vesicles, as discussed previously (30). The increase of donor lipid in the inner monolayer with D/A for both systems indicates that this is related to the CD-mediated exchange process. To obtain a measure for the degree of asymmetry, we define $\Sigma_{as} = \chi_{don}^{out} - \chi_{don}^{in}$, where $\chi_{don}^{out,\ in}$ are the mole fractions of donor lipid in the outer and inner bilayer leaflets, respectively. The resulting values show small differences for the two different D/A ratios for both systems. This suggests that all systems display a similar degree of asymmetry. The agreement of lipid composition determined by UPLC-MS on independently prepared aLUVs also shows good sample reproducibility.

Stability of asymmetric vesicles

Due to the different melting temperatures of POPE and POPC, the stability of lipid asymmetry is of some concern, particularly due to increased lipid flip-flop in the phase transition region (28) and the differential area expansivities of the gel and fluid phases (41,49,50).

Our ^{1}H-NMR experiments revealed a 14% decrease of lipid asymmetry when incubated at 35°C for nearly 5 days (Table S1). When equilibrated at 10°C, the observed change of asymmetry was insignificant within the uncertainty of the measurement. All experiments (DSC, DLS, TEM, and WAXS) were performed within less than a day of sample preparation, and all SAXS/SANS experiments were completed after three days of sample preparation. Hence, we expect no significant changes of lipid distribution in our samples.

Stability was further assessed by DSC, a highly sensitive technique for detecting changes in lipid composition. Only small changes in the thermograms of three consecutive cooling scans were observed (Fig. S4), indicating that there was no significant lipid scrambling across the melting transition of aLUVs. We further performed cryo-TEM experiments on aLUVs incubated in the phase-transition regime. No evidence for vesicle invagination or rupture was observed (Fig. S5).

TABLE 1 Leaflet Composition of Studied aLUVs

Component	χ_{POPC}^{in}	χ_{POPE}^{in}	χ_{POPC}^{out}	χ_{POPE}^{out}	Σ_{as}
$POPC^{out}/POPE^{in}$ [a]	0.06[b] (0.10[c])	0.94[b] (0.90[c])	0.54[b] (0.68[b])	0.46[b] (0.32[b])	0.48[b] (0.58[c])
$POPC^{out}/POPE^{in}$ [d]	0.11[b]	0.89[b]	0.64[b]	0.36[b]	0.53[b]
$POPE^{out}/POPC^{in}$ [a]	1.00[b] (1.00[c])	0.00[b] (0.00[c])	0.40[b] (0.33[c])	0.60[b] (0.67[c])	0.60[b] (0.67[c])
$POPE^{out}/POPC^{in}$ [d]	0.81[b]	0.19[b]	0.24[b]	0.76[b]	0.57[b]

[a]D/A = 2.
[b]Leaflet component mole fraction determined using DSC for lipid exchange.
[c]Leaflet component mole fraction determined using UPLC-MS for lipid exchange.
[d]D/A = 3.

Size and morphology

Temperature-induced changes in $POPC^{out}/POPE^{in}$ aLUV size were measured by DLS. Data revealed a linear change of vesicle size between 5 and 35°C (Fig. 3 *A*). The polydispersity in turn did not exhibit any temperature dependency. In general, PDI increased from <0.1 for acceptor vesicles to PDI $\sim 0.1 - 0.2$ for aLUVs. The linear increase of vesicle size with temperature is interesting, since the melting transition of symmetric bilayers is usually associated with significant changes in lipid volume and area (see, e.g., (52)). Indeed, DLS measurements of POPE* LUVs showed vesicle-size changes consistent with a melting at $T_M = 22\ ^\circ C$, as determined by DSC (Fig. 3 *A*).

To gain further insight, we determined the surface area expansion coefficient, $\alpha_S^T = 1/S \times \partial S/\partial T$, assuming spherical vesicles with an outer surface area S. For POPE* LUVs, α_S^T is about two times smaller in the gel phase than in the fluid phase, with a significant jump in the melting region (Fig. 3 *A*). In turn, α_S^T monotonically increases throughout the studied temperature range for aLUVs, with an end value close to that of fluid POPE*. This indicates an overall fluid-like behavior of the aLUVs, a notion further substantiated by the excellent agreement of the lipid area expansion coefficient, $\alpha_{A_L}^T$, reported for fluid POPE from SANS/SAXS experiments (50).

Comparing POPE* and POPE/POPC* vesicle diameters on an absolute scale reveals an ~10% increase in vesicle size during lipid exchange. This could be due to a change in vesicle morphology, e.g., aLUVs could become non-spherical, or due to a residual osmotic pressure as a result of an imbalance in the concentration of NaCl between the inner- and outer-vesicle aqueous phases. To resolve this, we performed cryo-TEM experiments at selected temperatures. Data revealed a majority of spherical aLUVs at both low and high temperatures (Fig. 3 *B*). This is in contrast to the faceted shaped vesicles displayed by POPE* LUVs in the gel phase (Fig. S6 A) and previously reported gel-phase giant unilamellar vesicles (53).

To check the influence of NaCl osmotic imbalance, we prepared POPE* LUVs with a 25 mM NaCl core and

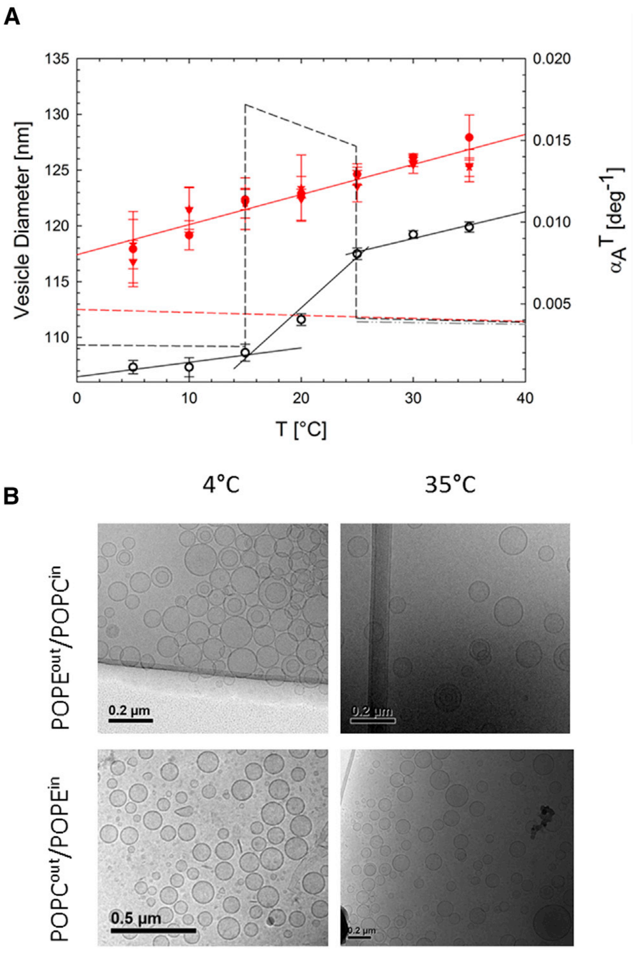

FIGURE 3 (*A*) Vesicle size (*symbols*) as a function of temperature and corresponding α_S^T, and $\alpha_{A_L}^T$ (*dashed lines*) for POPE^{out}/POPC^{in} (D/A = 2) aLUVs (*filled symbols*) and POPE* LUVs (*open symbols*), as determined from DLS. The dash-dotted gray line represents $\alpha_{A_L}^T$ values reported from scattering experiments (50). (*B*) Corresponding cryo-TEM images of POPE^{out}/POPC^{in} (*upper*) and POPC^{out}/POPE^{in} (*lower*) measured at 4°C (*left column*) and 35°C (*right column*). To see this figure in color, go online.

observed spherical vesicles by TEM (Fig. S6 B). The osmotic pressure resulting from NaCl in the core of aLUVs can be estimated by the Laplace equation, $\Delta P = 2\gamma/R$, where γ represents the surface tension and R is the vesicle radius. Using $R = 65$ nm and $\gamma = 41\ \mu$N/m (54), we calculate $\Delta P \simeq 0.01$ bar, which is too small to induce any detectable change to the nanoscopic leaflet structure—consistent with previous reports (30,33). However, defect lines in gel-phase vesicles can be expected to increase their flexibility, rendering them spherical even at low osmotic pressures.

Leaflet structure and thermotropic behavior

Melting of asymmetric leaflets

Phase transitions in POPE^{out}/POPC^{in} and POPC^{out}/POPE^{in} aLUVs were studied by DSC. Comparison of cooling scans from the two types of aLUVs reveal different behavior (Fig. 4). A single, but broad, melting transition was observed when POPE forms the inner leaflet. However, the melting of aLUVs with POPE comprising the major component in the outer leaflet is significantly broader, with two distinct melting transitions. Similar trends were observed for D/A = 2 aLUVs (Fig. S7).

It therefore seems that POPE^{out}/POPC^{out} aLUVs display an extended range of gel^{out}/fluid^{in} coexistence, whereas POPC^{out}/POPE^{in} aLUVs melt cooperatively, indicating strong interleaflet coupling. Moreover, the high-temperature melting transitions ($T_M^{PE/PC}$) of POPE^{out}/POPC^{in} aLUVs can be compared to the T_M for symmetric LUVs with the same outer-leaflet composition. For both D/A ratios, $T_M^{PE/PC}$ was $\sim 2-5\,°$C higher than the expected T_M. This indicates lipid domain formation (gel-fluid phase coexistence) within the outer monolayers, consistent with the occurrence of several heat capacity maxima—features

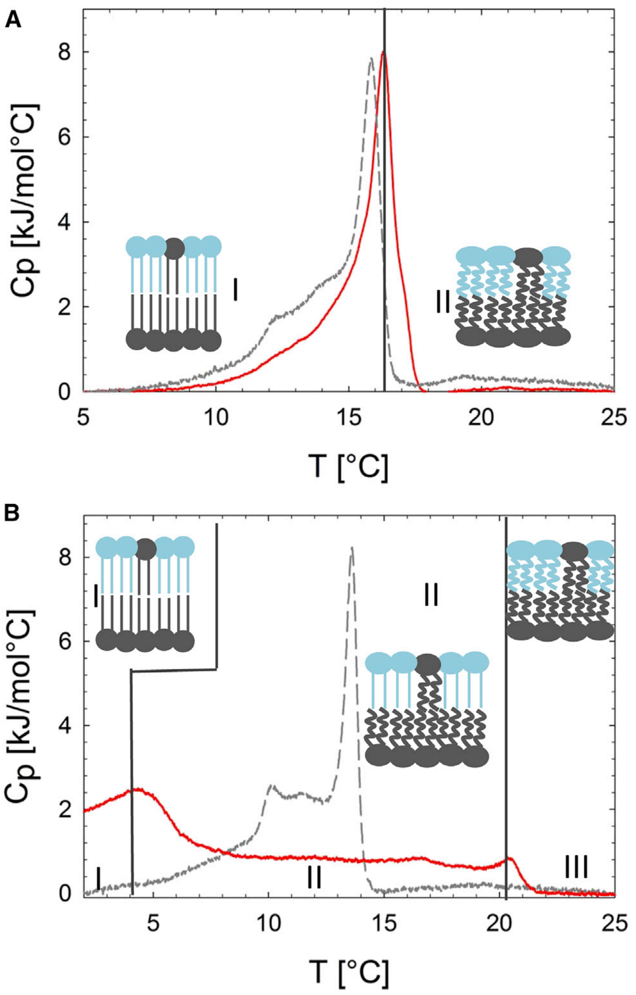

FIGURE 4 DSC cooling scans of POPC^{out}/POPE^{in} (*A*) and POPE^{out}/POPC^{in} (*B*) aLUVs (D/A = 3) (*solid lines*). Transitions of the corresponding scrambled LUVs are shown as gray dashed lines. (*Insets*) Schematics of leaflet structure. See Fig. S7 for the corresponding D/A = 2 data. To see this figure in color, go online.

that are particularly pronounced for aLUVs prepared at D/A = 2 (Fig. S7 B).

Lipid packing in gel-phase leaflets

Lipid lateral areas were determined from WAXS measurements. The most distinct difference in WAXS signals between aLUVs and scrambled LUVs was the width of the chain-chain correlation peak being much broader in the case of aLUVs (Fig. 5 *A*). This signifies a smaller gel-phase domain size for aLUVs, which can be also expressed in terms of the average chain-chain correlation length, ξ_D. In general, ξ_D ranged between 200 and 300 Å, and averaged over all temperatures, $\xi_D^{aLUV} < \xi_D^{LUV}$ (Fig. 5 *A*, *inset*). This is good evidence that gel-phase lipids are less well packed in aLUVs. Moreover, no additional peaks or shoulders were observed. Hence, acyl chain packing on a two-dimensional hexagonal lattice (with non-tilted hydrocarbons) is dominated by POPE in aLUVs, i.e., they form an L_β phase.

To determine the effect of lipid packing density in the bilayer leaflets, we compared the A_L values determined directly from WAXS of aLUVs to those calculated from their known monolayer composition (see Materials and Methods).

In the case of POPC^{out}/POPE^{in}, both leaflets form a gel phase in the temperature range shown in Fig. 5 *C*. Interestingly, theoretical A_L values show that lipids, on average, pack in aLUVs more tightly than in decoupled monolayers of equal outer-leaflet composition, but less so in decoupled monolayers of same inner-leaflet composition. That is, the observed lipid packing in aLUVs is a compromise between inner- and outer-leaflet lipids, and neither leaflet dominates over the other.

In the case of POPE^{out}/POPC^{in} aLUVs, WAXS data show coexistence of an inner POPC fluid leaflet and an outer leaflet dominated by POPE-enriched gel domains (Fig. 5 *B*). In particular, we observed an additional broad peak centered at $q \sim 13.8$ nm^{-1} , typical for hydrocarbons in the L_α phase (see, e.g., (55)). Note that fluid patches in the outer leaflet will also contribute to this peak. If we calculate the molecular averaged A_L using Eq. 5, according to the outer-leaflet composition and assuming that all POPC in the outer leaflet is in the gel phase, we find good agreement with experimental

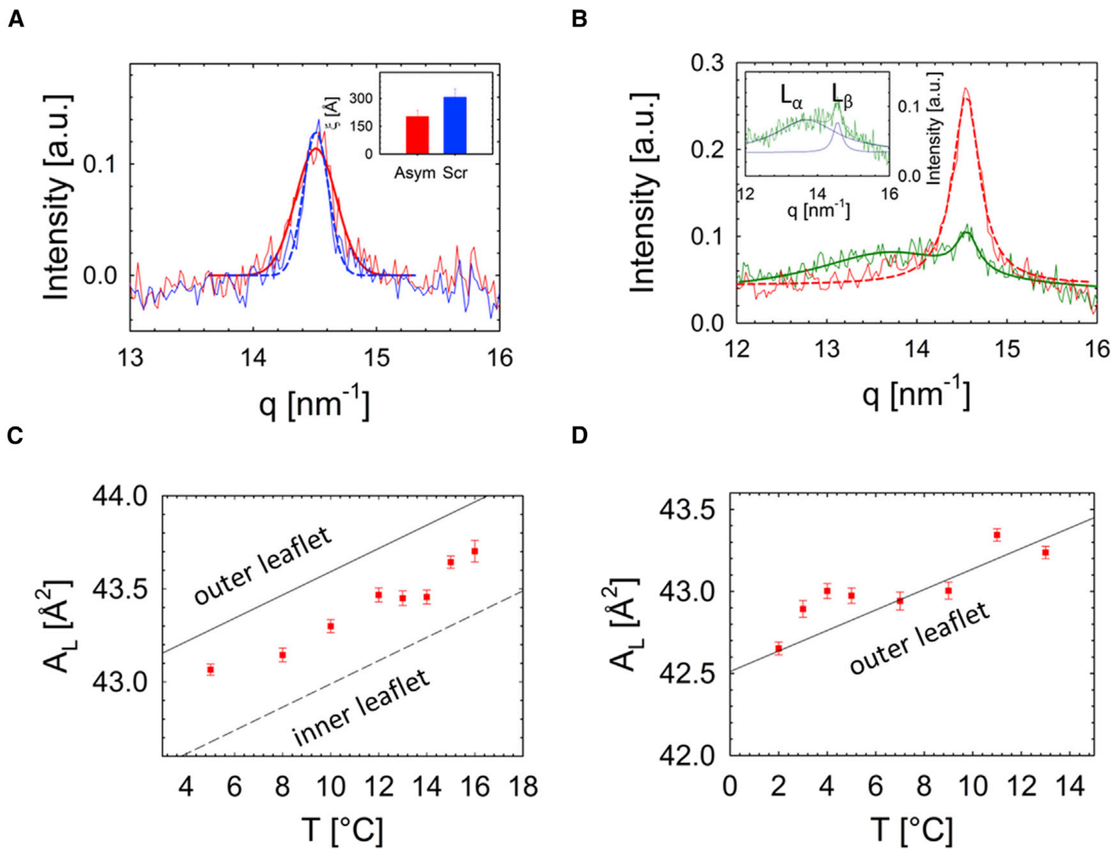

FIGURE 5 Wide-angle scattering and lipid packing of gel-phase aLUVs. (*A*) Comparison of WAXS data for aLUVs (*solid line*) and scrambled LUVs (*dashed line*) fitted by Gaussians. The inset compares the average χ_D of aLUVs and LUVs. (*B*) Comparison of POPE^{out}/POPC^{in} (*dashed line*; $T = 10°C$) and POPC^{out}/POPE^{in} (*solid line*; $T = 5°C$) WAXS data fitted by Gaussians. The POPE^{out}/POPC^{in} data contain an additional broad peak originating from fluid hydrocarbons (*inset*). (*C* and *D*) A_L values for POPC^{out}/POPE^{in} (*C*) and POPE^{out}/POPC^{in} aLUVs (*D*) as a function of temperature (*symbols*). Solid lines correspond to theoretical A_L values of the outer leaflet and the dashed line to theoretical A_L values of the inner leaflet calculated for a given leaflet composition. See Fig. S8 for the corresponding D/A = 2 data. To see this figure in color, go online.

data (Fig. 5 *D*). This provides additional proof that the two leaflets are not coupled. Note that if one assumes that POPC in the outer leaflet forms fluid domains, the calculated A_L value would decrease by ~0.2 Å^2. In reality, however, the amount of fluid POPC changes in the temperature range studied, and this is beyond the scope of this study.

Lipid packing in all-fluid leaflets

In the fluid phase, acyl chain-chain correlations are weak, and WAXS data do not allow for an unambiguous analysis of lipid packing in each leaflet. We therefore applied a joint analysis of SANS/SAXS data as detailed in the Materials and Methods.

Three scattering contrast conditions were analyzed for POPC^{out}/POPE^{in} aLUVs and their scrambled analogs (Figs. 6 and S9). Application of the aSDP model yielded reasonable agreement with experimental data. Note that these fits were constrained by lipid compositions detailed in Table 1. Deviations between fits and data are observed for the first scattering-intensity minimum. This "lift-off" may have several origins and can, for example, be accounted for assuming a small variation in membrane thickness. Although the introduction of an additional parameter to describe a thickness distribution resulted in a better fit, it did not affect the final structural parameters. We therefore chose to use the simpler model for the analysis presented here. For details of the obtained parameters, see Table S2. A_L values are reported in Table 2 and show, on average, a lower packing density of lipids in the outer leaflet compared to those in the inner leaflet (ΔA_L ~ 4 Å^2). However, this relates to the leaflet's lipid composition, as demonstrated by the A_L values calculated from molecular averages of pure POPE (50), POPC (49), and POPG (56) (Table 2). The remarkable agreement between measured and calculated A_L values shows that the lipid areas in each leaflet result from averaging the molecular packing properties of POPE and POPC lipids, and not from adjusting to the lipid packing in the apposing leaflet. Analysis of POPE^{out}/POPC^{in} aLUVs yielded comparable results (Fig. S10; Table 2), with the difference that the average packing of lipids in the inner leaflet is less dense than those in the outer leaflet. The good agreement with the calculated A_L value again demonstrates that the structural differences between the two leaflets relate to their compositional differences and not to any transbilayer coupling mechanism. Further, ΔA_L was similar for POPC^{out}/POPE^{in} aLUVs. Consequently, we conclude that fluid leaflets POPC^{out}/POPE^{in} and POPE^{out}/POPC^{in} aLUVs behave independently of each other. We note, however, that the experimental uncertainty of the WAXS data analysis (<0.1%) is significantly smaller than that of the joint SANS/SAXS data fits (<3%). Moreover, the observed changes in lipid packing densities of gel phase lipids due to the transbilayer coupling is <1% (Fig. 5). Hence, subtle features of transbilayer coupling in all-fluid POPE/POPC aLUVs may not be detectable.

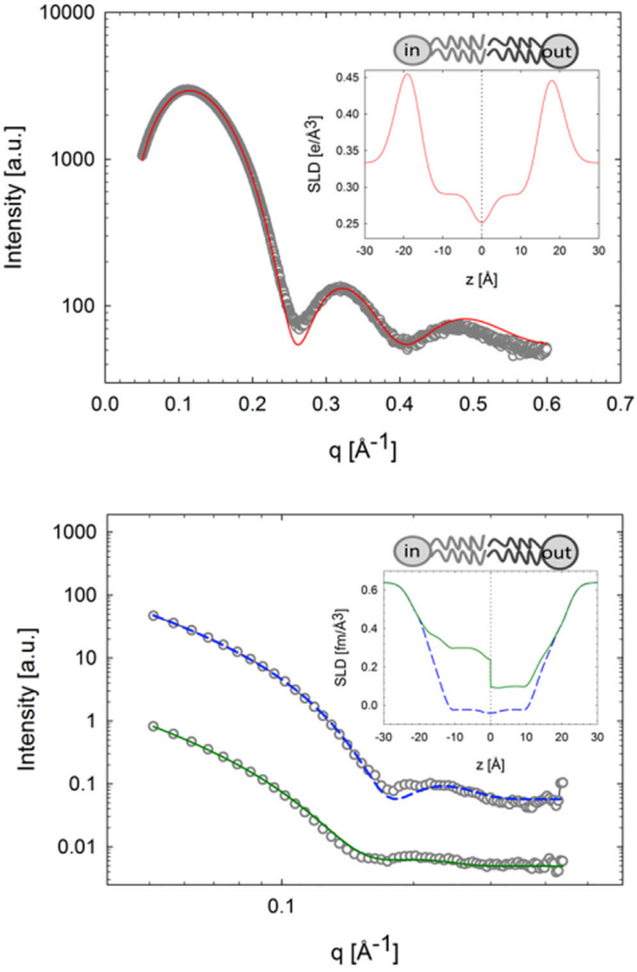

FIGURE 6 Joint analysis of SAXS (*upper*) and SANS (*lower*) data for POPC^{out}/POPE^{in} aLUVs at 35°C. Solid lines show best fits using the aSDP model (*dashed line*, POPC^{out}/POPE^{in}; *solid line*, POPC^{out}/POPE-d31^{in}). (*Insets*) Corresponding electron and neutron scattering-length density profiles. To see this figure in color, go online.

DISCUSSION

We studied POPC^{out}/POPE^{in} and POPE^{out}/POPC^{in} aLUVs over a range of temperatures using a complementary array of experimental techniques. This resulted in a comprehensive picture of membrane structure, from the microscopic to nanoscopic length scales. Cryo-TEM and DLS reported on the overall morphology and size of the vesicles, whereas

TABLE 2 Leaflet-Specific Lipid Areas of Fluid aLUVs

	A_L^{in} (Å^2)	A_L^{out} (Å^2)
POPC^{out}/POPE^{in}	59.7 (59.3[a])	64.7 (63.0[a])
POPE^{out}/POPC^{in}	64.7 (64.9[a])	59.9 (60.7[a])

D/A = 2. Experimental uncertainties are within 3%.
[a]Values calculated from leaflet composition using data reported in (49–51).

DSC combined with WAXS and SAXS/SANS experiments yielded insight into layer-specific structural details.

We found significant inter-leaflet coupling in POPC^{out}/POPE^{in} aLUVs in the gel phase. In the case of fluid POPC^{out}/POPE^{in} aLUVs, and also for POPE^{out}/POPC^{in} aLUVs at all temperatures, no transbilayer coupling was detected (Fig. 7). We will first discuss POPC^{out}/POPE^{in} at low temperatures. Here, our DSC experiments showed a single transition peak around 16.5°C (Fig. 4), indicating cooperative melting of the two leaflets. Our WAXS data analysis demonstrated that this coupling leads to a partial fluidization of the inner leaflet combined with a more densely packed outer leaflet, as compared to uncoupled leaflets of same lipid composition (Fig. 5). The average lipid packing in the outer and inner leaflets of this system therefore appears to be a compromise between the individual-leaflet properties. Interestingly, lateral positional correlations between acyl chains were less evident in aLUVs when compared to their symmetric counterparts (Fig. 5), suggesting an increase in defects in aLUVs (Fig. 7 *A*).

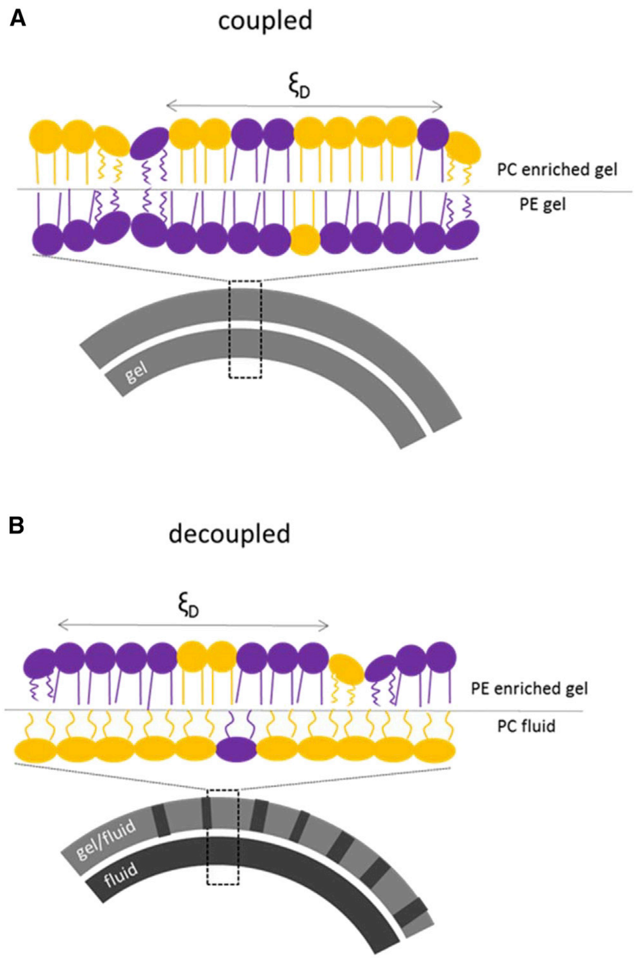

FIGURE 7 Schematic lipid distribution and transbilayer coupling in POPC^{out}/POPE^{in} (*A*) and POPE^{out}/POPC^{in} aLUVs (*B*). To see this figure in color, go online.

In the case of POPE^{out}/POPC^{in} aLUVs (Fig. 7 *B*), bilayer leaflets melted independently (Fig. 4), and acyl chain packing of gel domains in the outer leaflet resembled a normal L_β phase—also in terms of A_L values (Fig. 5, *B* and *D*). This is in contrast to our previous observations for DPPC^{out}/POPC^{in} aLUVs at low temperatures, where a significant disordering of the gel-like domains in the outer leaflet was observed, whereas the inner POPC layer did not differ from a pure POPC bilayer in terms of structure (30). Hence, POPE^{out}/POPC^{in} leaflets are uncoupled even at low temperatures. Due to the different lateral expansivities of the gel and fluid phases, this leads to significant strain within the vesicle, which may result in aLUV invagination or even rupture with increasing temperature. Area expansion is particularly pronounced across the melting transition, with an area increase of ~ 16 %, whereas a fluid membrane over the same temperature range expands by only 5% (estimated from WAXS data and literature (49,50)). However, TEM experiments yielded no evidence of significant morphological changes to the vesicles in the phase-transition region (Fig. S5), consistent with the high aLUV stability seen in repeated DSC scans (Fig. S4) and the slow lipid flip-flop (Table S1). Instead, our DLS data showed a fluid-like expansion of the vesicle surface area over the temperature range studied, including the gel-fluid coexistence region (Fig. 3 *A*). This can be rationalized by taking into account the increased number of defects present in the gel-phase regions of the outer leaflet, as evidenced in a smaller ξ_D, compared to symmetric vesicles (Fig. 5 *A*). Further, the fact that c_P never reaches baseline (Figs. 4 *B* and S7 B) indicates that melting of gel domains in the outer leaflet occurs continuously throughout the gel-fluid coexistence regime. We therefore speculate that gel-domain melting at the boundaries of the defect zones occurs with similar high expansivities, as observed in the phase transition regime of POPE* LUVs (Fig. 3 *A*). This would yield an overall fluid-like aLUV expansion, which avoids any vesicle-shape changes across the melting region.

Mechanisms of transbilayer coupling have been described using molecular models (partial hydrocarbon chain interdigitation, cholesterol flip-flop (14,15)), and continuum models (intrinsic curvature, electrostatic coupling, and entropic membrane undulations (12–16)). Due to the significant negative J_0 of POPE, as compared to POPC (57), an intrinsic curvature-mediated coupling appears to be the most likely explanation for the current system. This would energetically favor placing POPE in the inner leaflet of lipid vesicles. This can be further tested by changing the aLUV size, as the effect should be more pronounced for highly curved small vesicles, and would eventually disappear at larger vesicle sizes—this will be the subject of future work.

Interestingly, the POPE J_0 value decreases almost twice as fast with temperature relative to that of POPC (57). The intrinsic curvature strain stored within the aLUVs should

therefore increase with temperature, leading to an expected pronounced inter-leaflet coupling in the fluid phase. Yet, regardless of the location of POPE, fluid aLUVs do not exhibit any signs of coupling (Table 2).

This can be explained by considering the lipid acyl chain structure. POPE is a monounsaturated lipid, which means that its oleoyl chain is kinked even when in the gel phase and occupies an area greater than all- *trans* palmitic chains. This feature is also expressed by its A_L, which is $\sim 3 - 4$ Å^2 larger than that of dipalmitoyl phosphatidylethanolamine (35,58,59). Hence, even gel-phase POPE can be expected to display a significantly negative J_0. Additionally, lipids in the gel phase experience decreased motional entropy. That is, intrinsic curvature strain is less easily compensated by hydrocarbon chain dynamics. The latter effect may explain the absence of leaflet coupling in the fluid phase. Alternatively, experimental limitations in determining the A_L of fluid-phase bilayers with an accuracy comparable to that achieved in gel-phase bilayers may also explain this result. Technical developments capable of addressing this issue are currently taking place in our laboratories. We further note that the presence of POPG might affect the observed dependencies on POPE sidedness. Due to its negligible J_0 value, POPG should reduce the average inner-leaflet intrinsic curvature for POPC^{out}/POPE^{in} by ~ 10 %, and could therefore potentially cause a small decrease in transbilayer coupling strength. One might also expect that the differences in bending rigidities of gel and fluid bilayers (for review, see, e.g., (60)) could contribute to leaflet coupling. However, this should not depend on a specific enrichment of PE in one of the bilayer's leaflets, and it would not explain the distinct differences between POPC^{out}/POPE^{in} and POPE^{out}/POPC^{in}. Moreover, elastic strain energy depends linearly on bending rigidity but quadratically on J_0 (61). Hence, J_0 is more of a factor than membrane flexibility in affecting leaflet-specific strain energies.

Finally, the observed coupling for POPC^{out}/POPE^{in} may be influenced by the greater intermolecular hydrogen-bonding abilities of PE lipids (62). Differences in concentration of POPE in the inner/outer leaflets of POPC^{out}/POPE^{in} and POPE^{out}/POPC^{in} (Table 1) keep us from completely ruling out such contributions.

CONCLUSIONS

We presented experimental evidence for curvature-mediated coupling in gel-phase POPC^{out}/POPE^{in} aLUVs, which emphasizes the inner leaflet as being the energetically preferred location of POPE in plasma membranes (1–4). Interestingly, this leaflet coupling was not observed in the physiologically relevant fluid phase. This finding is in agreement with our recent report on DPPC^{out}/POPC^{in} aLUVs (33) and aLUVs enriched in SM in the outer leaflet (31,63). That is, the structure of fluid membranes is dominated by layer-specific membrane properties and is not influenced by that of the apposing leaflet. Consequently, hydrocarbon chain interdigitation (14,23), even if present, does not provide a sufficiently strong impetus to influence the two bilayer leaflets. However, noting that hydrocarbon chain asymmetry was recently reported to influence lipid diffusion but not the order of an apposing leaflet (32), we cannot exclude or comment on any effects on the lipid's lateral mobility, though we are planning to address this issue in future work. Future experiments will also focus on the role of cholesterol in the mechanical coupling of fluid membranes. Cholesterol was deliberately excluded from this study to keep the analysis tractable, but it is known to exchange rapidly between the two leaflets (64); it also has a significant negative intrinsic curvature (57), which may contribute to the coupling of fluid asymmetric membranes. The tools developed in the past couple of years (28,30,33) will allow us to address these issues in detail.

SUPPORTING MATERIAL

Supporting Materials and Methods, ten figures, and two tables are available at http://www.biophysj.org/biophysj/supplemental/S0006-3495(17)31238-9.

AUTHOR CONTRIBUTIONS

B.E. designed and performed the research, analyzed the data, and wrote the article. D.M. and F.A.H. designed and performed research. I.L.-P. performed TEM measurements. G.N.R. performed UPLC-MS experiments. M.-S.A. performed SANS measurements. J.K. gave conceptual advice and revised the manuscript. G.P. designed and performed research and wrote the article.

ACKNOWLEDGMENTS

We wish to thank Milka Doktorova, Sebastian Fiedler, and Heiko Heerklotz for helpful discussions, and, in particular, Erwin London for carefully reading the manuscript and for his numerous comments. We further thank Klaus Zangger and Bernd Werner (NMR, University of Graz), Chris Stanley (SANS-Oak Ridge National Laboratory (ORNL)), and Haydyn Mertens (SAXS-European Molecular Biology Lab/DESY) for technical assistance.

B.E. and G.P. were supported by the Austrian Science Fund FWF, project no. P27083-B20. J.K. and F.A.H. are supported through the Scientific User Facilities Division of the U.S. Department of Energy (DOE), Office of Basic Energy Sciences (BES), under contract no. DE-AC05 00OR2275. Research conducted at ORNL's Spallation Neutron Source was sponsored by the Scientific User Facilities Division, BES, U.S. DOE. ORNL is managed by UT-Battelle, LLC, under U.S. DOE contract no. DE-AC05-00OR22725. This work is based upon experiments performed at the KWS-1 instrument operated by the Jülich Centre for Neutron Science (JCNS) at the Heinz Maier-Leibnitz Zentrum (MLZ), Garching, Germany. The authors gratefully acknowledge the financial support provided by JCNS to perform the neutron scattering measurements at the MLZ. The research leading to these results has received funding from the European Communitys Seventh Framework Programme (FP7) under BioStruct-X (grant agreement N 283570).

SUPPORTING CITATIONS

References (65,66) appear in the Supporting Material.

REFERENCES

1. Verkleij, A. J., P. H. Ververgaert, …, P. F. Elbers. 1972. Phase transitions of phospholipid bilayers and membranes of *Acholeplasma laidlawii* B visualized by freeze fracturing electron microscopy. *Biochim. Biophys. Acta.* 288:326–332.
2. Bretscher, M. S. 1972. Asymmetrical lipid bilayer structure for biological membranes. *Nat. New Biol.* 236:11–12.
3. Devaux, P. F., and R. Morris. 2004. Transmembrane asymmetry and lateral domains in biological membranes. *Traffic.* 5:241–246.
4. Marquardt, D., B. Geier, and G. Pabst. 2015. Asymmetric lipid membranes: towards more realistic model systems. *Membranes (Basel).* 5:180–196.
5. Marrink, S. J., A. H. de Vries, …, S. R. Wassall. 2008. Cholesterol shows preference for the interior of polyunsaturated lipid membranes. *J. Am. Chem. Soc.* 130:10–11.
6. Mondal, M., B. Mesmin, …, F. R. Maxfield. 2009. Sterols are mainly in the cytoplasmic leaflet of the plasma membrane and the endocytic recycling compartment in CHO cells. *Mol. Biol. Cell.* 20:581–588.
7. Liu, S.-L., R. Sheng, …, W. Cho. 2017. Orthogonal lipid sensors identify transbilayer asymmetry of plasma membrane cholesterol. *Nat. Chem. Biol.* 13:268–274.
8. Simons, K., and D. Toomre. 2000. Lipid rafts and signal transduction. *Nat. Rev. Mol. Cell Biol.* 1:31–39.
9. Feigenson, G. W. 2009. Phase diagrams and lipid domains in multicomponent lipid bilayer mixtures. *Biochim. Biophys. Acta.* 1788:47–52.
10. Marsh, D. 2009. Cholesterol-induced fluid membrane domains: a compendium of lipid-raft ternary phase diagrams. *Biochim. Biophys. Acta.* 1788:2114–2123.
11. Wang, T. Y., and J. R. Silvius. 2001. Cholesterol does not induce segregation of liquid-ordered domains in bilayers modeling the inner leaflet of the plasma membrane. *Biophys. J.* 81:2762–2773.
12. Leibler, S., and D. Andelman. 1987. Ordered and curved meso-structures in membranes and amphiphilic films. *J. Phys. (Paris).* 48:2013–2018.
13. Shlomovitz, R., and M. Schick. 2013. Model of a raft in both leaves of an asymmetric lipid bilayer. *Biophys. J.* 105:1406–1413.
14. Collins, M. D. 2008. Interleaflet coupling mechanisms in bilayers of lipids and cholesterol. *Biophys. J.* 94:L32–L34.
15. May, S. 2009. Trans-monolayer coupling of fluid domains in lipid bilayers. *Soft Matter.* 5:3148–3156.
16. Galimzyanov, T. R., P. I. Kuzmin, …, S. A. Akimov. 2017. Undulations drive domain registration from the two membrane leaflets. *Biophys. J.* 112:339–345.
17. Kiessling, V., J. M. Crane, and L. K. Tamm. 2006. Transbilayer effects of raft-like lipid domains in asymmetric planar bilayers measured by single molecule tracking. *Biophys. J.* 91:3313–3326.
18. Wan, C., V. Kiessling, and L. K. Tamm. 2008. Coupling of cholesterol-rich lipid phases in asymmetric bilayers. *Biochemistry.* 47:2190–2198.
19. Collins, M. D., and S. L. Keller. 2008. Tuning lipid mixtures to induce or suppress domain formation across leaflets of unsupported asymmetric bilayers. *Proc. Natl. Acad. Sci. USA.* 105:124–128.
20. Polley, A., S. Mayor, and M. Rao. 2014. Bilayer registry in a multicomponent asymmetric membrane: dependence on lipid composition and chain length. *J. Chem. Phys.* 141:064903.
21. Perlmutter, J. D., and J. N. Sachs. 2011. Interleaflet interaction and asymmetry in phase separated lipid bilayers: molecular dynamics simulations. *J. Am. Chem. Soc.* 133:6563–6577.
22. Blosser, M. C., A. R. Honerkamp-Smith, …, S. L. Keller. 2015. Transbilayer colocalization of lipid domains explained via measurement of strong coupling parameters. *Biophys. J.* 109:2317–2327.
23. Horner, A., S. A. Akimov, and P. Pohl. 2013. Long and short lipid molecules experience the same interleaflet drag in lipid bilayers. *Phys. Rev. Lett.* 110:268101.
24. Stottrup, B. L., S. L. Veatch, and S. L. Keller. 2004. Nonequilibrium behavior in supported lipid membranes containing cholesterol. *Biophys. J.* 86:2942–2950.
25. Crane, J. M., and L. K. Tamm. 2004. Role of cholesterol in the formation and nature of lipid rafts in planar and spherical model membranes. *Biophys. J.* 86:2965–2979.
26. Crane, J. M., V. Kiessling, and L. K. Tamm. 2005. Measuring lipid asymmetry in planar supported bilayers by fluorescence interference contrast microscopy. *Langmuir.* 21:1377–1388.
27. Garg, S., J. Rühe, …, C. A. Naumann. 2007. Domain registration in raft-mimicking lipid mixtures studied using polymer-tethered lipid bilayers. *Biophys. J.* 92:1263–1270.
28. Marquardt, D., F. A. Heberle, …, G. Pabst. 2017. ^{1}H NMR shows slow phospholipid flip-flop in gel and fluid bilayers. *Langmuir.* 33:3731–3741.
29. Cheng, H. T., Megha, and E. London. 2009. Preparation and properties of asymmetric vesicles that mimic cell membranes: effect upon lipid raft formation and transmembrane helix orientation. *J. Biol. Chem.* 284:6079–6092.
30. Heberle, F. A., D. Marquardt, …, G. Pabst. 2016. Subnanometer structure of an asymmetric model membrane: interleaflet coupling influences domain properties. *Langmuir.* 32:5195–5200.
31. Cheng, H. T., and E. London. 2011. Preparation and properties of asymmetric large unilamellar vesicles: interleaflet coupling in asymmetric vesicles is dependent on temperature but not curvature. *Biophys. J.* 100:2671–2678.
32. Chiantia, S., and E. London. 2012. Acyl chain length and saturation modulate interleaflet coupling in asymmetric bilayers: effects on dynamics and structural order. *Biophys. J.* 103:2311–2319.
33. Eicher, B., F. A. Heberle, …, G. Pabst. 2017. Joint small-angle x-ray and neutron scattering data analysis of asymmetric lipid vesicles. *J. Appl. Crystallogr.* 50:419–429.
34. Kingsley, P., and G. W. Feigenson. 1979. The synthesis of a perdeuterated phospholipid: 1,2-dimyristoyl-*sn*-glycero-3-phosphocholine-d72. *Chem. Phys. Lipids.* 24:135–147.
35. Pozo Navas, B., K. Lohner, …, G. Pabst. 2005. Composition dependence of vesicle morphology and mixing properties in a bacterial model membrane system. *Biochim. Biophys. Acta.* 1716:40–48.
36. Pabst, G., A. Hodzic, …, P. Laggner. 2007. Rigidification of neutral lipid bilayers in the presence of salts. *Biophys. J.* 93:2688–2696.
37. Knittelfelder, O. L., B. P. Weberhofer, …, G. N. Rechberger. 2014. A versatile ultra-high performance LC-MS method for lipid profiling. *J. Chromatogr. B Analyt. Technol. Biomed. Life Sci.* 951-952:119–128.
38. Hartler, J., M. Trötzmüller, …, G. G. Thallinger. 2011. Lipid data analyzer: unattended identification and quantitation of lipids in LC-MS data. *Bioinformatics.* 27:572–577.
39. Petoukhov, M. V., D. Franke, …, D. I. Svergun. 2012. New developments in the ATSAS program package for small-angle scattering data analysis. *J. Appl. Crystallogr.* 45:342–350.
40. McIntosh, T. J., and S. A. Simon. 1986. Area per molecule and distribution of water in fully hydrated dilauroylphosphatidylethanolamine bilayers. *Biochemistry.* 25:4948–4952.
41. Marsh, D. 2013. Handbook of Lipid Bilayers, Second Edition. CRC Press, Boca Raton, FL.
42. Blanchet, C. E., A. Spilotros, …, D. I. Svergun. 2015. Versatile sample environments and automation for biological solution X-ray scattering experiments at the P12 beamline (PETRA III, DESY). *J. Appl. Crystallogr.* 48:431–443.

43. Petoukhov, M. V., P. V. Konarev, …, D. I. Svergun. 2007. ATSAS 2.1—towards automated and web-supported small-angle scattering data analysis. *J. Appl. Crystallogr.* 40:s223–s228.

44. Frielinghaus, H., A. Feoktystov, …, G. Mangiapia. 2015. KWS-1: small-angle scattering diffractometer. *J. Large-Scale Res. Facilities.* 1:A28.

45. Feoktystov, A. V., H. Frielinghaus, …, T. Brüeckel. 2015. KWS-1 high-resolution small-angle neutron scattering instrument at JCNS: current state. *J. Appl. Crystallogr.* 48:61–70.

46. Arnold, O., J. C. Bilheux, …, J. Zikovsky. 2014. Mantid—data analysis and visualization package for neutron scattering and μSR experiments. *Nucl. Instrum. Methods Phys. Res.* 764:156–166.

47. Kiselev, M. A., P. Lesieur, …, V. L. Aksenov. 2002. Model of separated form factors for unilamellar vesicles. *Appl. Phys., A Mater. Sci. Process.* 74 (*Suppl.1*):s1654–s1656.

48. Pencer, J., V. N. P. Anghel, …, J. Katsaras. 2006. Scattering from laterally heterogeneous vesicles. I. Model-independent analysis. *J. Appl. Crystallogr.* 39:791–796.

49. Kučerka, N., M. P. Nieh, and J. Katsaras. 2011. Fluid phase lipid areas and bilayer thicknesses of commonly used phosphatidylcholines as a function of temperature. *Biochim. Biophys. Acta.* 1808:2761–2771.

50. Kučerka, N., B. van Oosten, …, J. Katsaras. 2015. Molecular structures of fluid phosphatidylethanolamine bilayers obtained from simulation-to-experiment comparisons and experimental scattering density profiles. *J. Phys. Chem. B.* 119:1947–1956.

51. Pan, J., X. Cheng, …, J. Katsaras. 2014. The molecular structure of a phosphatidylserine bilayer determined by scattering and molecular dynamics simulations. *Soft Matter.* 10:3716–3725.

52. Heimburg, T. 1998. Mechanical aspects of membrane thermodynamics. Estimation of the mechanical properties of lipid membranes close to the chain melting transition from calorimetry. *Biochim. Biophys. Acta.* 1415:147–162.

53. Pataraia, S., Y. Liu, …, R. Dimova. 2014. Effect of cytochrome *c* on the phase behavior of charged multicomponent lipid membranes. *Biochim. Biophys. Acta.* 1838:2036–2045.

54. Editorial Committee. 1995. Tables of Physical & Chemical Constants, 16th edition (originally compiled by G. W. C. Kaye and T. H. Laby, 1918), 2.2.5 Surface tensions. www.kayelaby.npl.co.uk.

55. Boulgaropoulos, B., M. Rappolt, …, G. Pabst. 2012. Lipid sorting by ceramide and the consequences for membrane proteins. *Biophys J.* 102:2031–2038.

56. Pan, J., D. Marquardt, …, J. Katsaras. 2014. Revisiting the bilayer structures of fluid phase phosphatidylglycerol lipids: accounting for exchangeable hydrogens. *Biochim. Biophys. Acta.* 1838:2966–2969.

57. Kollmitzer, B., P. Heftberger, …, G. Pabst. 2013. Monolayer spontaneous curvature of raft-forming membrane lipids. *Soft Matter.* 9:10877–10884.

58. McIntosh, T. J. 1980. Differences in hydrocarbon chain tilt between hydrated phosphatidylethanolamine and phosphatidylcholine bilayers. A molecular packing model. *Biophys. J.* 29:237–245.

59. Lohner, K., A. Latal, …, P. Garidel. 2001. Packing characteristics of a model system mimicking cytoplasmic bacterial membranes. *Chem. Phys. Lipids.* 111:177–192.

60. Dimova, R. 2014. Recent developments in the field of bending rigidity measurements on membranes. *Adv. Colloid Interface Sci.* 208:225–234.

61. Helfrich, W. 1973. Elastic properties of lipid bilayers: theory and possible experiments. *Z. Naturforsch. C.* 28:693–703.

62. Boggs, J. M. 1980. Intermolecular hydrogen bonding between lipids: influence on organization and function of lipids in membranes. *Can. J. Biochem.* 58:755–770.

63. Chiantia, S., P. Schwille, …, E. London. 2011. Asymmetric GUVs prepared by MβCD-mediated lipid exchange: an FCS study. *Biophys. J.* 100:L1–L3.

64. Bennett, W. F. D., J. L. MacCallum, …, D. P. Tieleman. 2009. Molecular view of cholesterol flip-flop and chemical potential in different membrane environments. *J. Am. Chem. Soc.* 131:12714–12720.

65. Andrews, S. B., J. W. Faller, …, R. J. Barrnett. 1973. Lanthanide ion-induced isotropic shifts and broadening for nuclear magnetic resonance structural analysis of model membranes. *Proc. Natl. Acad. Sci. USA.* 70:1814–1818.

66. Heimburg, T. 2007. Thermal Biophysics of Membranes. Wiley-VCH, Berlin, Germany.

Biophysical Journal, Volume *114*

Supplemental Information

Intrinsic Curvature-Mediated Transbilayer Coupling in Asymmetric Lipid Vesicles

Barbara Eicher, Drew Marquardt, Frederick A. Heberle, Ilse Letofsky-Papst, Gerald N. Rechberger, Marie-Sousai Appavou, John Katsaras, and Georg Pabst

Supplementary Material

Instrinsic Curvature-Mediated Transbilayer Coupling in Asymmetric Lipid Vesicles

B. Eicher, D. Marquardt, F.A. Heberle, I. Letofsky-Papst, G.N. Rechberger, M.-S. Appavou, J. Katsaras, and G. Pabst

1 Sample preparation

Acceptor vesicles were prepared in 25 mM NaCl solution to a lipid concentration of 10 mg/mL. All acceptor vesicles were doped with 10 mol% POPG or POPG-d31 matching the isotopic composition of the inner leaflet hydrocarbons. Doping vesicles with PG facilitates LUV formation (see below). Throughout this work we report POPE/POPC* molar ratios, only, where the asterisk indicates the presence of POPG. During hydration samples were incubated for one hour at $\geq$ 10°C above the lipid's melting transition (T_M) with intermittent vortex mixing, followed by 5 freeze/thaw cycles of the hydrated vesicles using liquid nitrogen. Finally, LUVs were obtained by passing the lipid dispersions 31 times ($T > T_M + 10$°C) through 100 nm pore-diameter polycarbonate filters using a hand-held mini-extruder (Avanti Polar Lipids, Alabaster, AL, USA). In turn, donor vesicles (composed of outer leaflet lipids) were prepared in the form of multilamellar vesicles (MLVs) by hydrating the dry films in 20% (w/w) aqueous sucrose solution at a lipid concentration of 20 mg/mL, followed by an 1-hour incubation at $T > T_M + 10$°C, with intermittent vortex mixing, and 3 freeze/thaw cycles. Finally, donor MLVs were diluted by a factor of 20 with water and centrifuged for 30 min at 20,000 $\times g$ to remove extravesicular sucrose.

Lipid exchange was initiated by re-suspending the pellet containing donor vesicles in 35 mM mβCD (lipid:mβCD = 1:8) followed by a 2 h incubation at room temperature while being gently stirred. Next, acceptor LUVs were added to the mβCD/donor solution and stirred gently for 30 min ($T > T_M$+10°C for POPE acceptor vesicles and T = room temperature for POPC acceptor vesicles). Two different donor/acceptor molar ratios (D/A = 2 and D/A = 3) were applied yielding a different lipid exchange (see below). Dispersions containing the final aLUVs were diluted by a factor of seven with water and centrifuged at 20,000 $\times g$ for 30 min. The supernatant (containing aLUVs, residual CD and sucrose) was removed carefully and concentrated with a centrifugal ultrafiltration device (100 kDa cutoff) to <0.5 mL. Finally, sucrose and CD were removed by repeated washing in either D_2O for ^{1}H-NMR and SANS experiments, or in H_2O for all other experiments.

For control experiments, vesicles with the same but symmetric lipid distribution as aLUVs were dried down to a film under reduced atmospheric pressure using a rotary evaporator (Heidolph, Germany) with the water bath set to 40°C. The dried lipid film was redissolved in chloroform and from that point on prepared as acceptor vesicles including the extrusion step. The resulting LUVs are called 'scrambled' vesicles throughout this report.

2 Evaluation of bilayer asymmetry via ^{1}H-NMR

The paramagnetic lanthanide ion Pr^{3+} interacts with choline protons, shifting their resonance downfield as shown in (1), see Fig. S2. By adding Pr^{3+} to a vesicle suspension, the shift is selective for outer leaflet protiated choline, leading to a separate resolution of the protiated choline resonances from the inner and outer leaflet (2). The integrated area R of each resonance is proportional to the number of molecules having protiated headgroups in the corresponding leaflet (Fig. S2). The outer leaf's peak fraction is defined as:

$$f^{out} = \frac{R^{out}}{R^{in} + R^{out}}, \tag{1}$$

where the superscripts 'out' and 'in' indicate the outer and inner leaflet. When all lipids posses protiated headgroups, f^{out} directly yields the mole fraction of all bilayer lipids found in the outer leaflet:

$$X^{out} = \frac{\sum_j N_j^{out}}{\sum_j N_j} \equiv f^{out}, \tag{2}$$

where N and N^{out} denote the number of molecules in the whole bilayer and in the outer leaflet and the summation is performed over all components of the mixture. For a bilayer with an equal number of lipids in the leaflets $X^{out} = 0.5$. However, POPE and POPC have different A_L (3, 4) which means that X^{out} is defined by the area per lipid of the inner (A_L^{in}) and of the outer leaflet (A_L^{out}):

$$X^{out} = \frac{1/A_L^{out}}{1/A_L^{out} + 1/A_L^{in}} * 1.06, \tag{3}$$

where the factor 1.06 considers the slightly bigger surface of the outer to the inner leaflet (5) by assuming a vesicle size of 130 nm and a bilayer thickness of 40 Å. As only one mixture component possesses a protiated choline (POPC) we define the single-component outer leaflet peak fraction f_{PC}^{out} as:

$$f_{PC}^{out} = \frac{N_{PC}^{out}}{N_{PC}} = \frac{X^{out}\chi_{PC}^{out}}{\chi_{PC}}, \tag{4}$$

where χ_{PC}^{out} stands for the outer leaflet mole fraction of POPC. Combining the two previous equations gives the following expression for the outer leaflet mole fraction of POPC:

$$\chi_{PC}^{out} = \frac{f_{PC}^{out}\chi_{PC}}{f_{PC}^{out}}. \tag{5}$$

For a two component POPC/POPE bilayer, all compositional parameters $\chi_{PC/PE}^{out,in}$ can be expressed as:

$$\begin{array}{lcl} \chi_{PC}^{out} & = & \frac{f_{PC}^{out}\chi_{PC}}{X^{out}} \\ \chi_{PC}^{in} & = & \frac{(1-f_{PC}^{out})\chi_{PC}}{(1-X^{out})} \\ \chi_{PE}^{in(out)} & = & 1 - \chi_{PC}^{in(out)} \end{array}. \tag{6}$$

3 Supplementary Figures

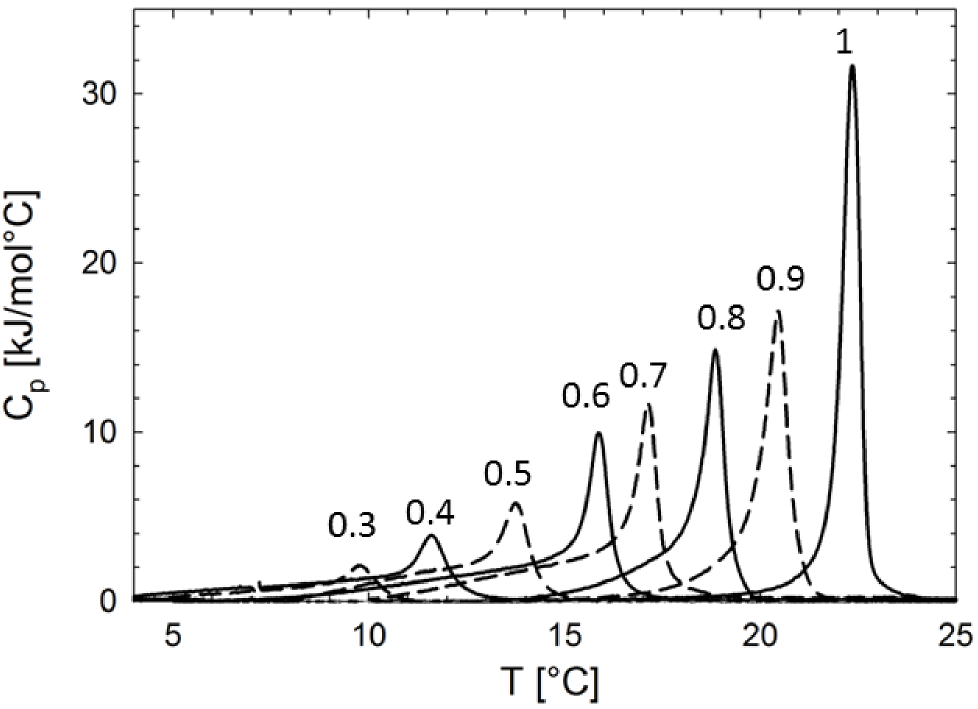

Figure S1: DSC cooling thermograms of POPE/POPC* MLV mixtures (numbers adjacent to data give the molar fractions of POPE). Note that compared to LUV data (Fig. 1) no solidus peak is visible. This relates to subtle differences in melting of LUVs and MLVs. Due to geometric constraints MLVs typically exhibit a significantly higher cooperative melting transition (see e.g. (6)). The solidus peak observed for LUVs indicates phase separation. The absence of this peak in MLV data consequently suggests that these phases are not coupled across the interstitial water layers. Note that these differences do not affect the position of the liquidus peak. DSC scans on MLVs and LUVs yield within experimental error identical T_M's (Fig. 1, insert), signifying the robustness of the applied calibration.

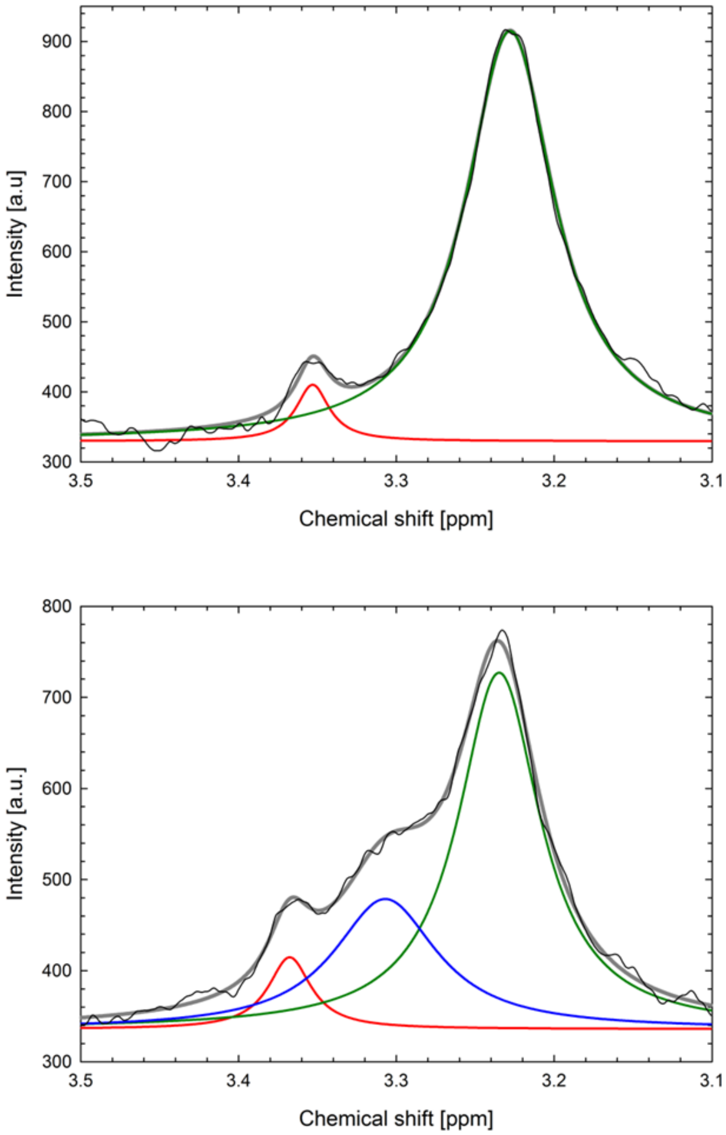

Figure S2: *Upper panel*: ^{1}H-NMR shows the choline resonance (green Lorentzian) from POPC acceptor lipids, while the red Lorentzian considers the contribution of residual mβCD. *Lower panel*: ^{1}H-NMR signal in the presence of the shift reagent Pr^{3+}. The shifted population (blue Lorentzian) relative to the unshifted population (green) reveals inner leaflet acceptor enrichment in the aLUVs.

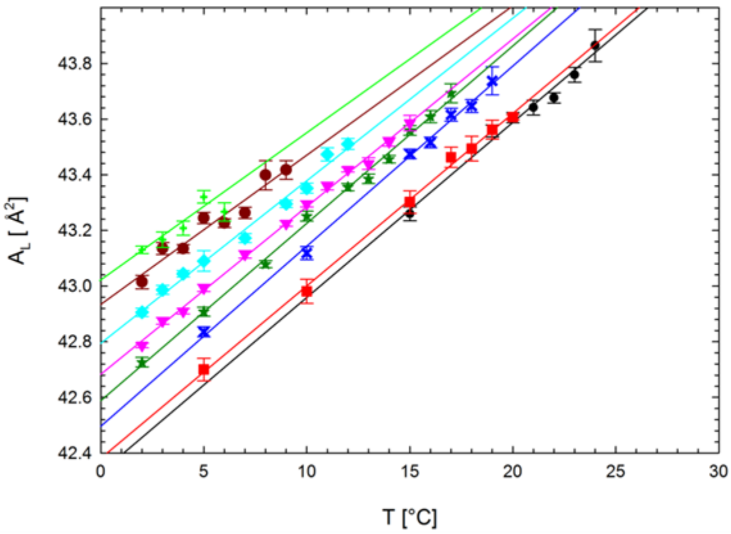

Figure S3: Temperature dependence of areas per lipid for different POPE/POPC* mixtures. The increase of POPC concentration leads to a progressive upshift of lipid areas (black line: $\chi_{POPE} = 1$, green line: $\chi_{POPE} = 0.3$)

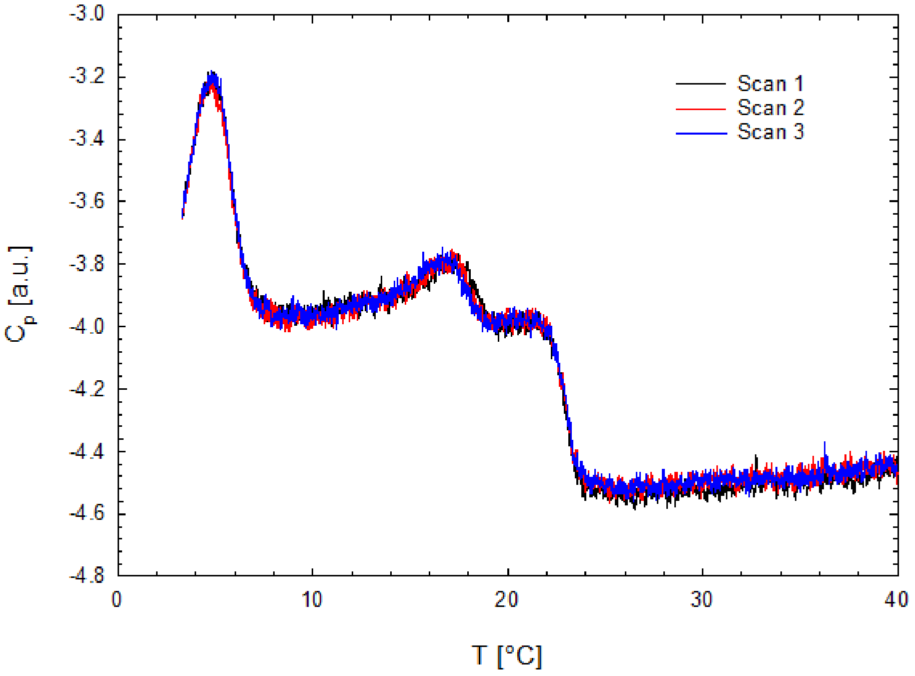

Figure S4: Three consecutive heating scans of POPE^{out}/POPC^{in} (D/A = 3). Data were background subtracted, but not normalized for sample concentration.

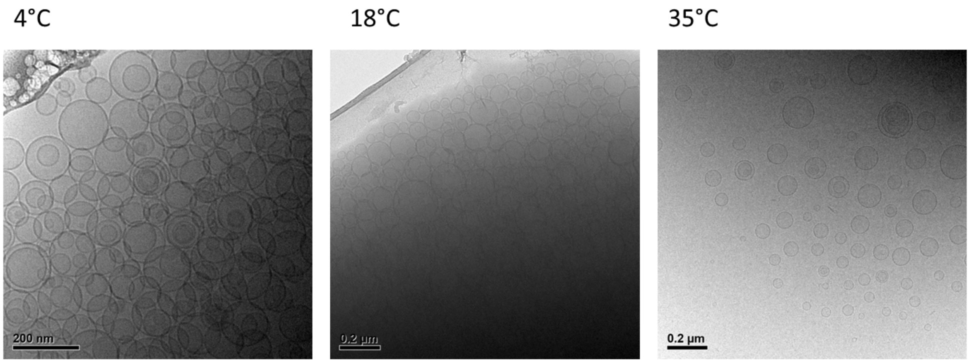

Figure S5: Cryo-TEM images of POPE^{out}/POPC^{in} aLUVs at different temperatures. The middle panel corresponds to the phase transition regime.

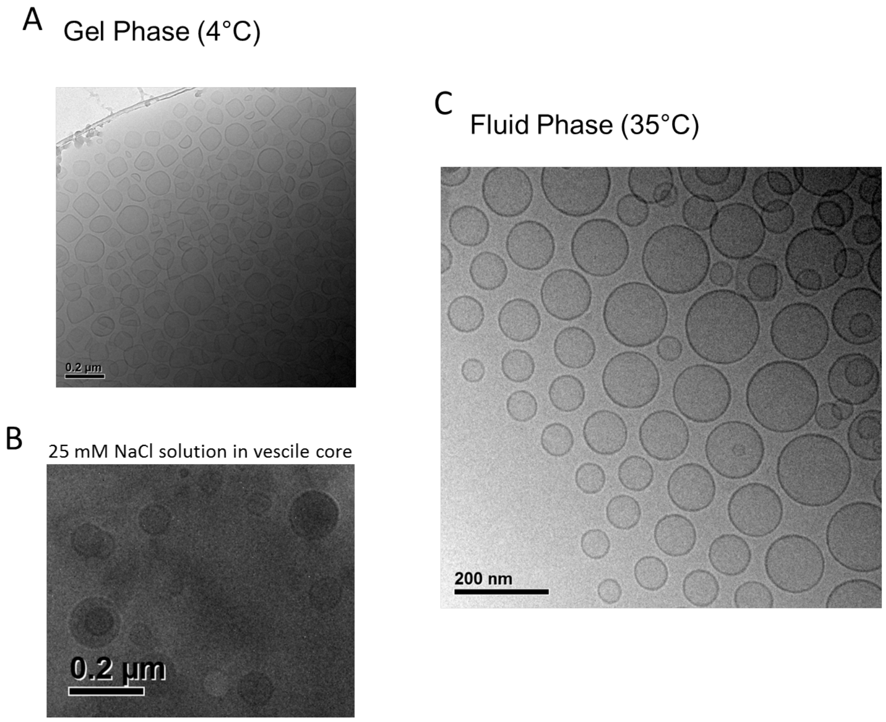

Figure S6: Cryo-TEM images of POPE* LUVs in the gel-phase without (panel A) and with a 25 mM NaCl core (panel B). Panel C shows the LUVs in fluid phase.

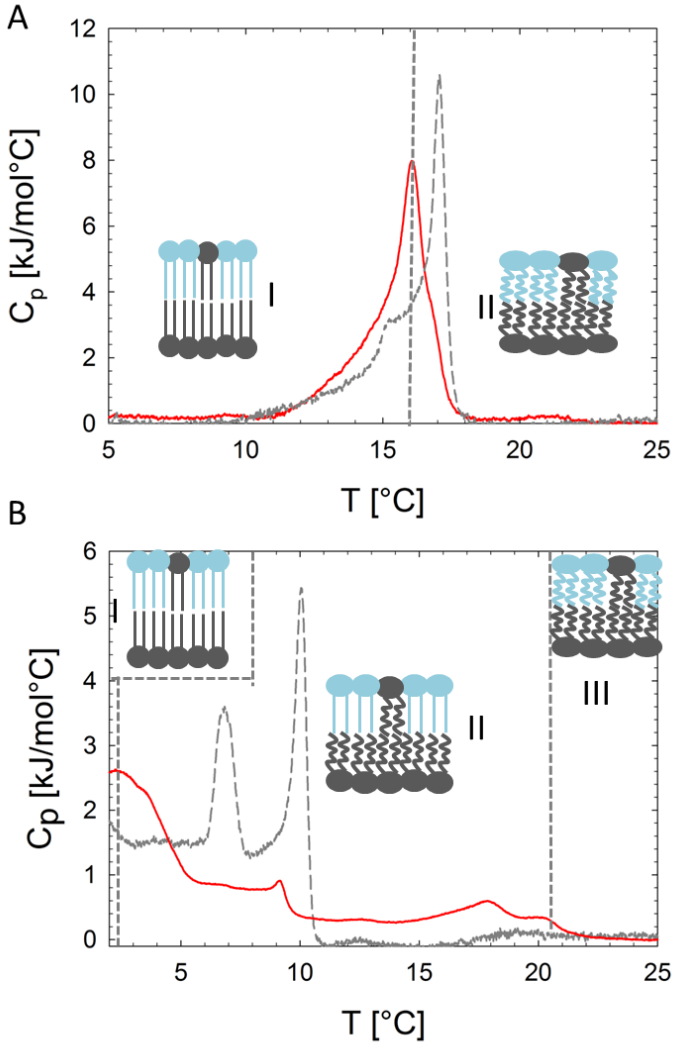

Figure S7: DSC cooling scans of POPC^{out}/POPE^{in} (panel A) and POPE^{out}/POPC^{in} (panel B) aLUVs (D/A = 2) (red lines). Transitions of corresponding scrambled LUVs are shown as gray dashed lines. Insets show schematics of leaflet structure.

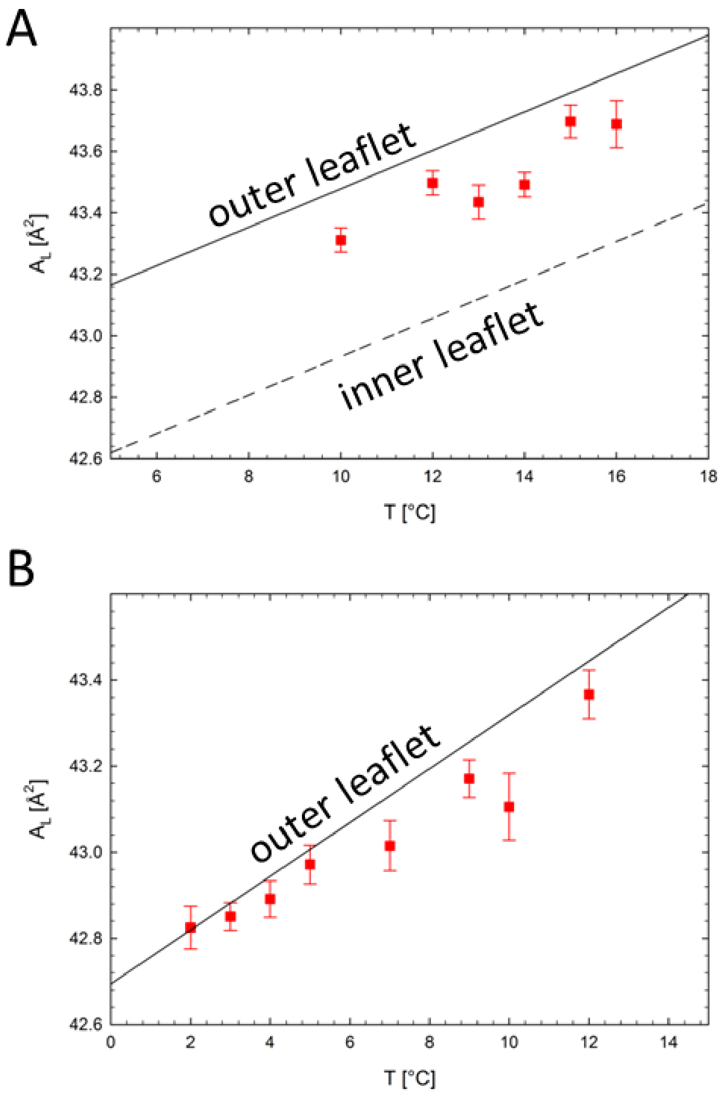

Figure S8: Area per lipid for POPC^{out}/POPE^{in} (A) and for POPE^{out}/POPC^{in} (B) as a function of temperature (symbols). Solid lines correspond to theoretical A_L's of the outer leaflet and the dashed line to theoretical A_L's of the inner leaflet calculated from the given leaflet composition.

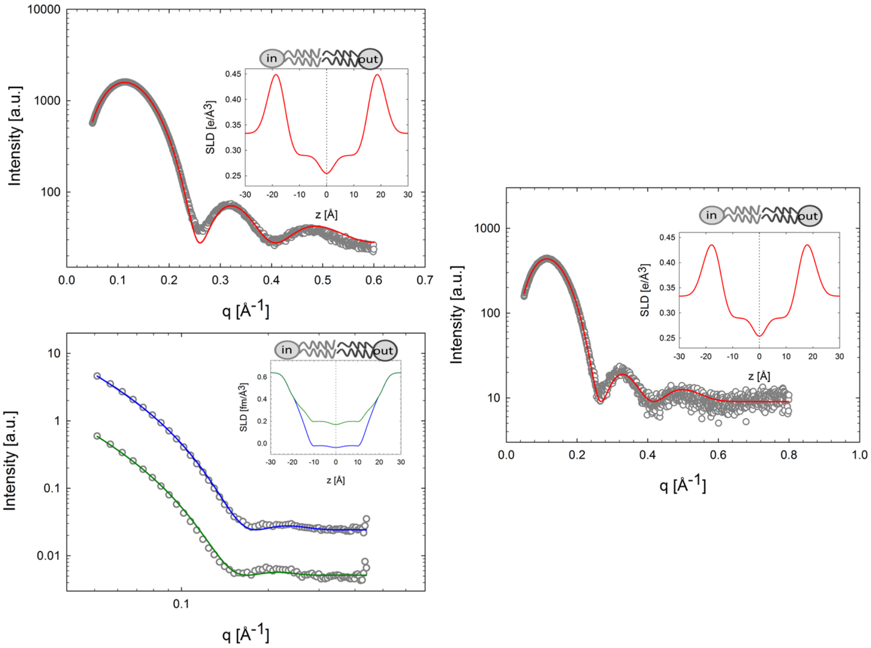

Figure S9: Analysis of scattering data of scrambled POPE/POPC LUVs (T = 35°C). *Left panel*: SAXS (*top*) and SANS (*lower*) data of POPC^{don}/POPE^{acc} LUVs. *Right panel*: SAXS data of POPE^{don}/POPC^{acc} LUVs. Solid lines correspond to best fits using the SDP model. SANS data have been obtained at two contrasts (blue line: POPC^{don}/POPE^{acc}, green line: POPC^{don}/POPE-d31^{acc}). Inserts show the corresponding scattering length density profiles.

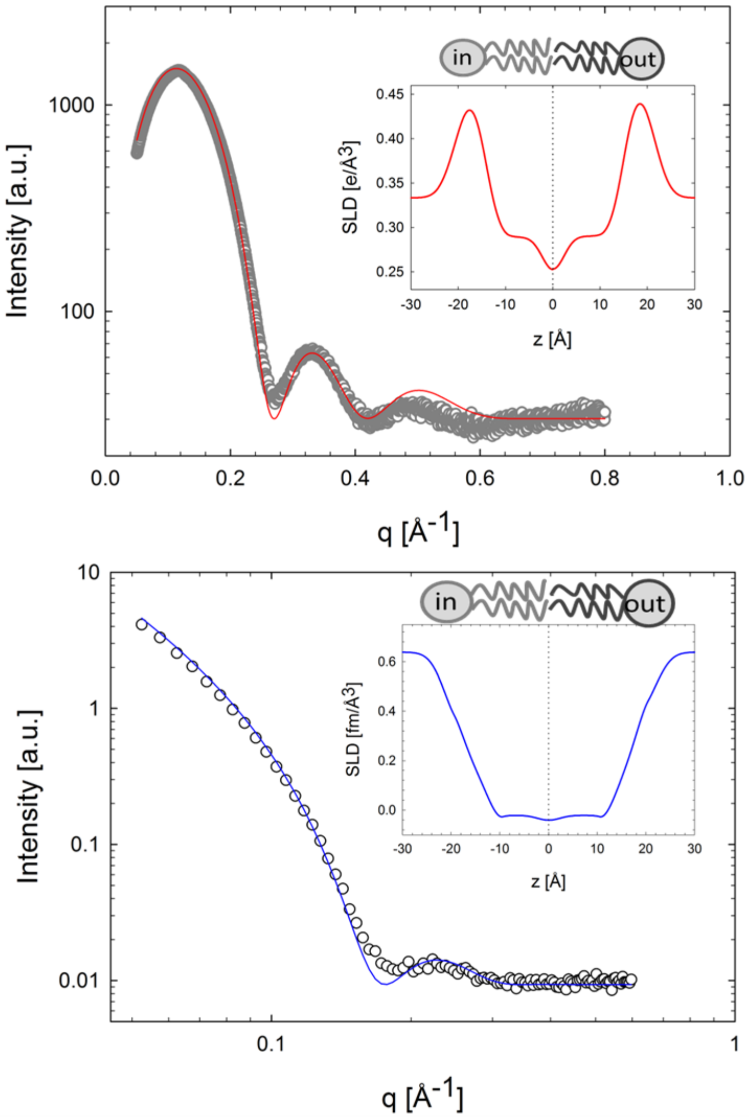

Figure S10: Joint analysis of SAXS (top panel) and SANS (lower panel) data of POPE^{out}/POPC^{in} aLUVs at 35°C. Solid lines show best fits using the aSDP model (blue line POPE^{out}/POPC^{in}). Inserts show the corresponding scattering length density profiles.

4 Supplementary Tables

Table S1: Decay of normalized bilayer asymmetry determined from ^{1}H-NMR.

	**POPE^{out}/POPC^{in}**		**POPC^{out}/POPE^{in}**	
	10℃	35℃	10℃	35℃
time (h)	ΔC	ΔC	ΔC	ΔC
0	1.00±0.04	1.00 ± 0.04	1.00 ± 0.09	1.00 ±0.08
20	1.07± 0.20	0.98 ± 0.04	1.00 ± 0.09	0.97 ± 0.07
70	1.00 ± 0.09	0.91 ± 0.05	0.94 ± 0.07	0.93 ± 0.08
118	0.96 ± 0.03	0.86 ± 0.03	0.98 ± 0.07	0.94 ± 0.05

Table S2: Structural parameters of asymmetric and scrambles POPC^{out}/POPE^{in} and POPE^{out}/POPC^{in} vesicles 35 °C determined with the aSDP-model.

	POPC^{don}/POPE^{acc}			POPE^{don}/POPC^{acc}		
	asym		scram	asym		scram
	out	in		out	in	
A_L [Å^2]	64.7	59.7	61.5	59.9	64.7	63.2
σ_{RH} [Å]	2.87	2.79	2.85	2.84	2.95	2.85
σ_{CG} [Å]	2.46	2.47	2.49	2.52	2.51	2.45
σ_M [Å]	2.00	1.99	2.04	2.03	2.01	1.97
σ_{MN} [Å]	5.01	5.01	5.01	5.01	5.01	4.95
$\|z_{RH}\|$ [Å]	19.62	20.04	19.69	20.13	18.92	19.36
$\|z_{CG}\|$ [Å]	16.35	17.56	17.12	17.02	15.88	16.21
$\|z_M\|^*$ [Å]	1.00	1.00	1.00	1.00	1.00	1.00
$\|z_{MN}\|$ [Å]	14.37	15.58	15.13	15.52	14.38	14.71

*fixed parameter

References

1. Heberle, F. A., D. Marquardt, M. Doktorova, B. Geier, R. F. Standaert, P. Heftberger, B. Kollmitzer, J. D. Nickels, R. A. Dick, G. W. Feigenson, J. Katsaras, E. London, and G. Pabst, 2016. Subnanometer Structure of an Asymmetric Model Membrane: Interleaflet Coupling Influences Domain Properties. *Langmuir* 32:5195–5200.

2. Andrews, S. B., J. W. Faller, J. M. Gilliam, and R. J. Barrnett, 1973. Lanthanide ion-induced

isotropic shifts and broadening for nuclear magnetic resonance structural analysis of model membranes. *Proc Natl Acad Sci USA* 1814–1818.

3. Kučerka, N., M. P. Nieh, and J. Katsaras, 2011. Fluid phase lipid areas and bilayer thicknesses of commonly used phosphatidylcholines as a function of temperature. *Biochim Biophys Acta* 1808:2761–2771.

4. Kučerka, N., B. van Oosten, J. Pan, F. A. Heberle, T. A. Harroun, and J. Katsaras, 2015. Molecular structures of fluid phosphatidylethanolamine bilayers obtained from simulation-to-experiment comparisons and experimental scattering density profiles. *J Phys Chem B* 119:1947–1956.

5. Marquardt, D., B. Geier, and G. Pabst, 2015. Asymmetric lipid membranes: towards more realistic model systems. *Membranes* 5:180–196.

6. Heimburg, T., 2007. Thermal Biophysics of Membranes. Wiley-VCH, Berlin.
